# Substrate-mediated regulation of the arginine transporter of *Toxoplasma gondii*

**DOI:** 10.1371/journal.ppat.1009816

**Published:** 2021-08-05

**Authors:** Esther Rajendran, Morgan Clark, Cibelly Goulart, Birte Steinhöfel, Erick T. Tjhin, Simon Gross, Nicholas C. Smith, Kiaran Kirk, Giel G. van Dooren

**Affiliations:** 1 Research School of Biology, Australian National University, Canberra, Australia; 2 School of Life Sciences, University of Technology Sydney, Sydney, Australia; University of South Florida, UNITED STATES

## Abstract

Intracellular parasites, such as the apicomplexan *Toxoplasma gondii*, are adept at scavenging nutrients from their host. However, there is little understanding of how parasites sense and respond to the changing nutrient environments they encounter during an infection. *Tg*ApiAT1, a member of the apicomplexan ApiAT family of amino acid transporters, is the major uptake route for the essential amino acid L-arginine (Arg) in *T*. *gondii*. Here, we show that the abundance of *Tg*ApiAT1, and hence the rate of uptake of Arg, is regulated by the availability of Arg in the parasite’s external environment, increasing in response to decreased [Arg]. Using a luciferase-based ‘biosensor’ strain of *T*. *gondii*, we demonstrate that the expression of *Tg*ApiAT1 varies between different organs within the host, indicating that parasites are able to modulate *Tg*ApiAT1-dependent uptake of Arg as they encounter different nutrient environments *in vivo*. Finally, we show that Arg-dependent regulation of *Tg*ApiAT1 expression is post-transcriptional, mediated by an upstream open reading frame (uORF) in the *Tg*ApiAT1 transcript, and we provide evidence that the peptide encoded by this uORF is critical for mediating regulation. Together, our data reveal the mechanism by which an apicomplexan parasite responds to changes in the availability of a key nutrient.

## Author summary

Intracellular parasites of the phylum Apicomplexa, including the opportunistic pathogen *Toxoplasma gondii*, are adept at scavenging nutrients from their host. Emerging evidence suggests that apicomplexans are able to sense and respond to changes in nutrient availability in their environment. These responses mediate important processes such as parasite virulence and progression into new stages of parasite life cycles. However, the mechanisms by which parasites sense and respond to nutrient availability are poorly understood. In the present study, we demonstrate that the expression of *Tg*ApiAT1, an essential, plasma membrane-localized arginine transporter that serves as the primary arginine uptake route in *T*. *gondii* parasites, is regulated by the arginine concentration that the parasite encounters in its environment, both *in vitro* and *in vivo*. We demonstrate that this regulation is mediated by an upstream open reading frame (uORF) in the 5’ untranslated region of the *Tg*ApiAT1 transcript, and that regulation is dependent on the sequence of the uORF-encoded peptide, one of only a few examples in nature where a uORF-encoded peptide has such a role. Our study provides an example of how *T*. *gondii* senses and responds to the nutrient status of its host, a phenomenon which may contribute to the extraordinarily broad host cell specificity exhibited by this parasite.

## Introduction

Apicomplexans are a phylum of intracellular parasites that include the causative agents of malaria (*Plasmodium* spp.) and toxoplasmosis (*Toxoplasma gondii*). The proliferation of parasites in their hosts, and their progression through their often-complex life cycles, is dependent on nutrients scavenged from the host [1–3]. Apicomplexans encounter different nutrient conditions as they proliferate within, and move between, hosts, and this is reflected in differences in the metabolism of different parasite life-stages; *e*.*g*., hepatocyte stages of *Plasmodium* parasites rely on the biosynthesis of heme and fatty acids, whereas the intra-erythrocytic parasite stages scavenge these from the host [4,5]. Although early studies suggested that parasite metabolism is ‘hard-wired’ and resistant to changes in nutrient conditions [6], there is growing evidence that parasites sense and respond to changes in the nutrient status of their hosts [3]. For example, *Plasmodium* blood-stage parasites modulate their proliferation in response to the caloric intake of their hosts, and can enter a dormant state in response to limitation of the essential amino acid isoleucine [7,8].

In some instances, the ability of parasites to sense changes in external nutrient levels is key to their differentiation into new life stages. For example, limitation of lysophosphatidylcholine induces *Plasmodium falciparum* parasites to differentiate into the transmitted sexual stages in the human host [9], and the high levels of linoleic acid that *T*. *gondii* parasites encounter in the intestines of felids induces parasite differentiation into the sexual stages [10]. The depletion of the amino acid arginine (Arg), which may be caused by host immune responses [11], is thought to lead to differentiation of the disease-causing tachyzoite stage of *T*. *gondii* into the dormant, cyst-forming bradyzoite stage [12]. Despite the importance of nutrient sensing in parasite proliferation and differentiation, the mechanisms by which parasites sense and respond to the availability of nutrients are largely unknown.

The uptake of nutrients by the disease-causing tachyzoite stage of *T*. *gondii* parasites is mediated primarily by plasma membrane transporters [13]. We recently characterised a family of plasma membrane amino acid transporters that are found throughout apicomplexans and have termed these the Apicomplexan Amino acid Transporter (ApiAT) family [14]. We have demonstrated that one member of this family, *Tg*ApiAT1 (www.toxodb.org gene identifier TGME49_215490), is an Arg transporter that is essential for *T*. *gondii* virulence [15].

In this study, we have investigated the ability of *T*. *gondii* parasites to sense and respond to the Arg levels that they encounter in their host. We report Arg-dependent regulation of *Tg*ApiAT1 expression, and demonstrate that this process is mediated by an upstream open reading frame (uORF) in the *Tg*ApiAT1 transcript. We also present evidence, obtained using a luciferase-based ‘biosensor’ strain of *T*. *gondii*, that parasites vary the expression of *Tg*ApiAT1 in different organs within their host. Our data demonstrate how *T*. *gondii* parasites are able to sense and respond to changes in the abundance of a key nutrient, as well as illustrating their ability to do so within the course of an infection.

## Results

### Regulation of *Tg*ApiAT1 protein abundance and parasite arginine uptake

**Fig 1:**
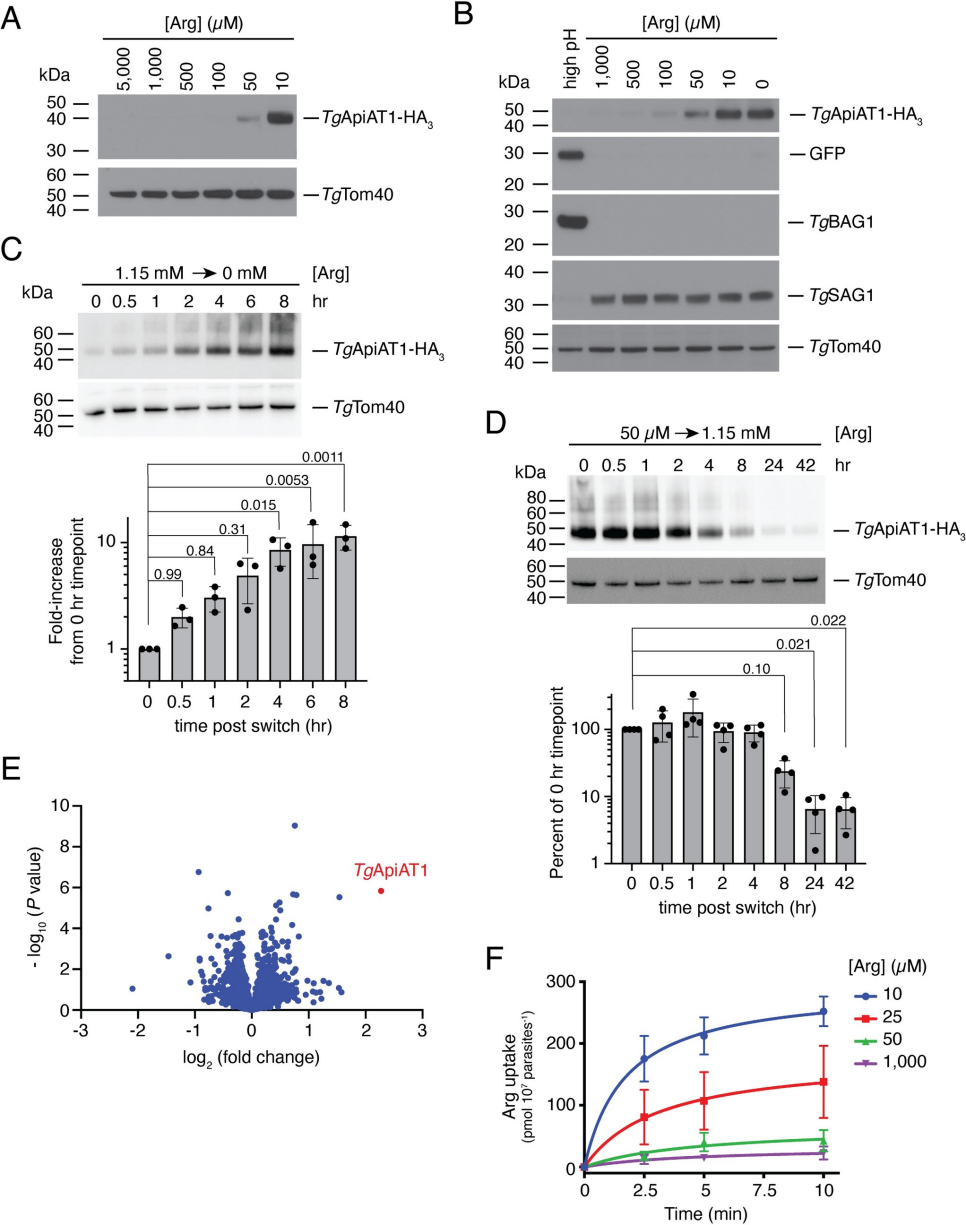
*Tg*ApiAT1 protein abundance is regulated by [Arg] in the growth medium. (**A**) Western blot of *Tg*ApiAT1-HA_3_ in parasites grown at a range of [Arg] in the growth medium. *Tg*Tom40 is a loading control. Data are representative of three independent experiments. (**B**) Western blot of *Tg*ApiAT1-HA_3_ in Prugniaud strain parasites co-expressing GFP from the bradyzoite-specific *Tg*LDH2 upstream region. Parasites were cultured at a range of [Arg] in the growth medium for 2 days, or at a high pH to induce bradyzoite formation for 6 days. The high pH induction medium contained 1.15 mM Arg. Western blots were probed with antibodies against anti-HA (to detect *Tg*ApiAT1-HA_3_), anti-GFP (to detect GFP expressed from the bradyzoite-specific promoter LDH2), anti-BAG1 (a bradyzoite-specific marker), anti-SAG1 (a tachyzoite-specific marker), and anti-*Tg*Tom40 (a loading control). (**C-D**) (top) Western blots detecting *Tg*ApiAT1-HA_3_ in parasites grown in medium containing high [Arg] (1.15 mM) and switched to medium lacking Arg (C), or parasites grown in medium containing low [Arg] (50 mM) and switched to medium containing high [Arg] (1.15 mM; D), for the indicated times. *Tg*Tom40 is a loading control. Data are representative of three (C) or four (D) independent experiments. (bottom) Quantification of the fold-increase in *Tg*ApiAT1-HA_3_ abundance upon the switch from high to zero [Arg] (C) or the percent decrease in *Tg*ApiAT1-HA_3_ abundance upon the switch from low to high Arg (D). Band intensities were normalised to the *Tg*Tom40 loading control. Values represent the mean ± SD from three (C) or four (D) independent experiments. *P* values were calculated using a one-way ANOVA with Dunnett’s multiple comparisons test, comparing each value to the 0 hr condition, with relevant *P* values depicted on the graphs. (**E**) Volcano plot depicting log_2_ fold change vs -log_10_ *P* values of change in protein abundance in a SWATH MS-based proteomic analysis of parasites grown at 50 μM vs 1.15 mM Arg (n = 5). The *Tg*ApiAT1 data point is depicted in red. (**F**) Timecourse of Arg uptake in parasites grown in medium containing a range of [Arg]. Parasites were cultured in growth medium containing 10, 25, 50 and 1,000 μM Arg for 2 days. Uptake was measured in the presence of 50 μM unlabelled Arg, 80 μM unlabelled Lys and 0.1 μCi/ml [^14^C]Arg, for 0 to 10 min. Data represent the mean ± s.e.m. from three independent experiments.

To investigate whether the expression of *Tg*ApiAT1 is dependent upon Arg availability, we introduced a haemagglutinin (HA_3_) epitope tag into the open reading frame of the *Tg*ApiAT1 genomic locus. The resultant *Tg*ApiAT1-HA_3_-expressing parasites were cultured for two days in modified Roswell Park Memorial Institute 1640 (RPMI) medium in which the starting concentration of Arg ranged from 10 μM to 5 mM. Western blotting revealed that the abundance of the major ~43 kDa species of *Tg*ApiAT1-HA_3_ varied with [Arg], with *Tg*ApiAT1-HA_3_ most abundant in parasites grown at low [Arg] (**Fig 1A**). In many anti-*Tg*ApiAT1-HA_3_ western blots, we observed additional minor higher molecular mass species which exhibited a similar Arg-dependent response (**S1 Fig**). These were not investigated further.

Low [Arg] conditions have been linked to formation of the latent bradyzoite stage of *T*. *gondii* [12]. We HA-tagged *Tg*ApiAT1 in Type II Prugniaud strain *T*. *gondii* parasites, which readily form bradyzoites, and cultured parasites for two days at a range of initial [Arg] in the culture medium. As for Type I parasites, expression of *Tg*ApiAT1-HA_3_ increased with decreasing [Arg] (**Fig 1B**). To test whether *Tg*ApiAT1-HA_3_ abundance changed upon bradyzoite differentiation, we induced bradyzoite differentiation by incubating parasites for six days in high pH conditions in a medium containing 1.15 mM Arg. We observed increased expression of the bradyzoite-specific antigen *Tg*BAG1 and a bradyzoite-specific green fluorescence protein (GFP), and decreased expression of the tachyzoite specific antigen *Tg*SAG1, verifying that bradyzoite differentiation had occurred (**Fig 1B**). Notably, we observed no induction of *Tg*ApiAT1-HA_3_ expression in these conditions (**Fig 1B**). These data indicate that *Tg*ApiAT1 regulation is not related to the general bradyzoite differentiation response of the parasite, and that Arg-dependent *Tg*ApiAT1 regulation occurs in both Type I and Type II strains of *T*. *gondii*. The short timeframe over which we cultured parasites in depleted [Arg] means that our data do not rule out the possibility that Arg depletion causes bradyzoite differentiation, or rule out a role for *Tg*ApiAT1 in mediating bradyzoite differentiation.

To assess the timeframe of the Arg-dependent regulation of *Tg*ApiAT1-HA_3_, we switched Type I strain *Tg*ApiAT1-HA_3_ parasites growing in medium containing 1.15 mM Arg to medium lacking Arg, then cultured the parasites in the Arg-free medium for a further 0.5 to 8 hr. We observed a rapid increase in *Tg*ApiAT1-HA_3_ abundance, with the protein level having increased eight-fold by four hours after the medium switch (**Fig 1C**). In a converse experiment, in which *Tg*ApiAT1-HA_3_ parasites were switched from medium containing 50 μM Arg to medium containing 1.15 mM Arg and cultured for a further 0.5 to 42 hr, decreases in *Tg*ApiAT1-HA_3_ protein levels were not observed until 8 hr after the switch, at which time *Tg*ApiAT1-HA_3_ abundance had decreased to 24 ± 10% (mean ± S.D., n = 4) of that in parasites cultured in 50 μM Arg, with a further decrease in *Tg*ApiAT1-HA_3_ abundance to 7 ± 4% (mean ± S.D., n = 4) of that in the non-switched parasites after 24 hr (**Fig 1D**). These data reveal that *T*. *gondii* parasites change the abundance of their major Arg transporter in response to the [Arg] they encounter in their growth medium in a timeframe of hours, with parasites able to upregulate *Tg*ApiAT1-HA_3_ expression in response to Arg limitation more rapidly than they are able to reduce *Tg*ApiAT1-HA_3_ protein levels when [Arg] becomes abundant.

To assess whether the abundance of other proteins changed upon changes to [Arg] in the growth medium, we cultured parasites in media containing either 50 μM or 1.15 mM Arg and extracted proteins for quantitative proteomics using sequential window acquisition of all theoretical fragment ion spectra mass spectrometry (SWATH-MS; [16]). This revealed that although some proteins exhibited changed abundances between the two conditions, only *Tg*ApiAT1 was upregulated by >four-fold in the 50 μM compared to the 1.15 mM condition (**Fig 1E** and **S1 Table**).

We next set out to establish whether changes in *Tg*ApiAT1 abundance induced by culturing parasites at different [Arg] correlate with changes in *Tg*ApiAT1-dependent Arg uptake by the parasite. *T*. *gondii* parasites have two Arg transporters: the selective Arg transporter *Tg*ApiAT1 and the general cationic amino acid transporter *Tg*ApiAT6-1, which has a higher affinity for L-lysine (Lys) than Arg [15,17]. It is possible to measure *Tg*ApiAT1-dependent Arg uptake by performing uptake assays in the presence of 80 μM Lys, under which condition Arg uptake by *Tg*ApiAT6-1 is inhibited [15]. Parasites were cultured at a range of [Arg] and the *Tg*ApiAT1-dependent uptake of [^14^C]-labelled Arg was measured in parasites suspended in media containing 80 μM Lys and 50 μM unlabelled Arg. We observed a concentration-dependent increase in *Tg*ApiAT1-dependent Arg uptake as the [Arg] in the culture medium decreased (**Fig 1F**). These data indicate that increased expression of *Tg*ApiAT1 resulting from depletion of Arg in the growth medium correlates with increased uptake of Arg into the parasite through this transporter.

### The 5’ region of the *Tg*ApiAT1 gene regulates *Tg*ApiAT1 protein abundance

**Fig 2:**
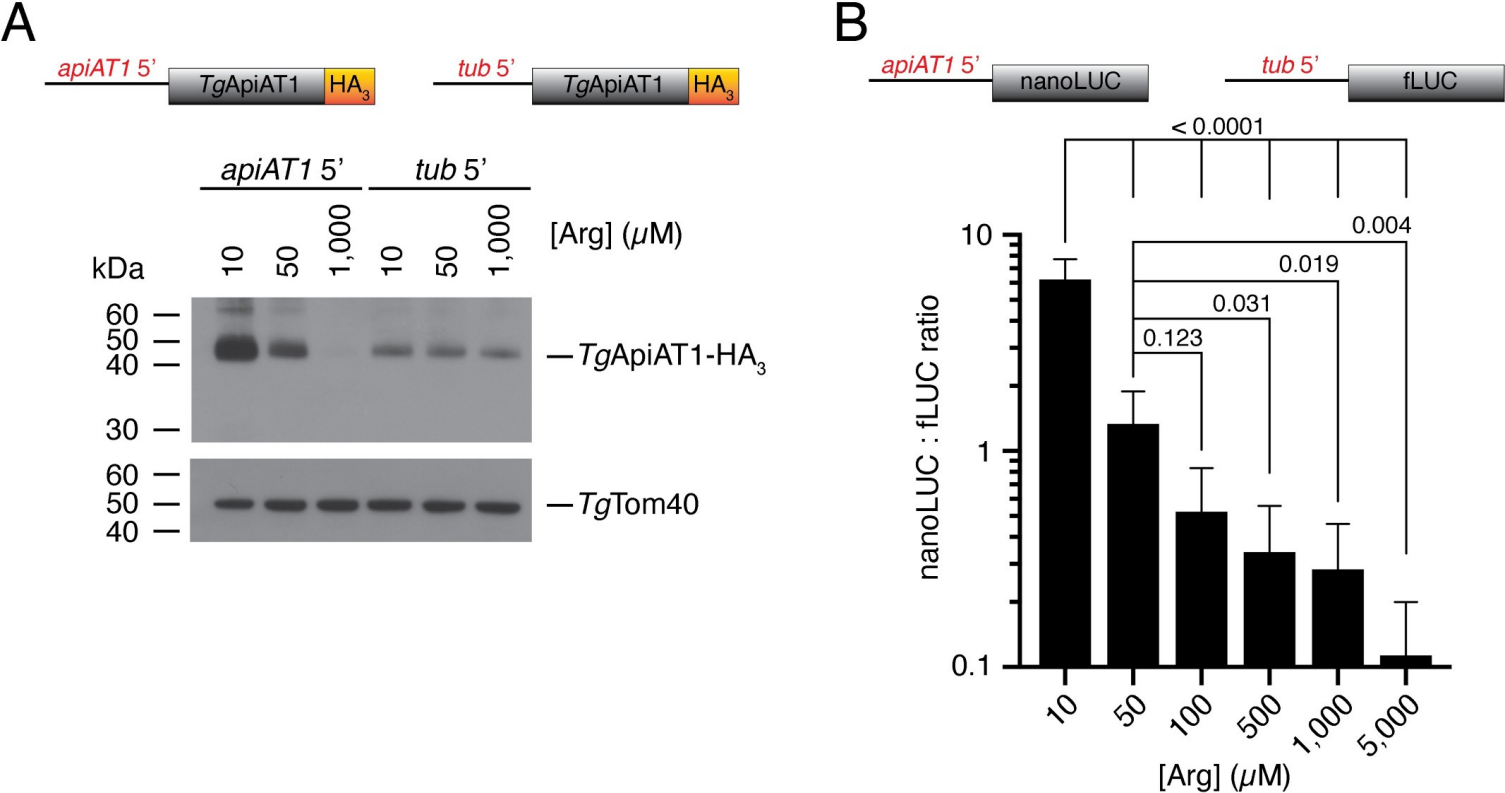
Arg-dependent *Tg*ApiAT1 regulation is mediated by the 5’ upstream region of the *Tg*ApiAT1 gene. (**A**) Western blot of *Tg*ApiAT1-HA_3_ expressed from the native *Tg*ApiAT1 5’ region (*apiAT1* 5’) or the α-tubulin 5’ region (*tub* 5’), in parasites grown at a range of [Arg] in the growth medium. *Tg*Tom40 is a loading control. Data are representative of three independent experiments. (**B**) nanoLUC:fLUC ratio in a parasite strain expressing nanoLUC from the *Tg*ApiAT1 5’ region (*apiAT1* 5’-nanoLUC) and fLUC from the α-tubulin 5’ region (*tub* 5’-fLUC), and grown at a range of [Arg] for 40–42 hr. Data represent the mean ± SD from nine independent experiments. *P* values were calculated using a one-way ANOVA with Tukey’s multiple comparisons test. *P* values not shown were > 0.50.

The expression of many proteins is mediated by genetic information encoded upstream (5’) of the start codon. To test whether the 5’ region of the gene encoding *Tg*ApiAT1 is important for regulation, we measured *Tg*ApiAT1-HA_3_ abundance in a strain in which *Tg*ApiAT1-HA_3_ was expressed from the α-tubulin promoter and in which the native *Tg*ApiAT1 gene had been knocked out [15]. We grew this strain at 10 μM, 50 μM and 1 mM Arg. Western blotting revealed no variation in *Tg*ApiAT1-HA_3_ abundance (**Fig 2A**), indicating that the 5’ region of the *Tg*ApiAT1 coding sequence is necessary for Arg-dependent regulation of *Tg*ApiAT1.

To determine whether the 5’ region of the gene encoding *Tg*ApiAT1 is sufficient to mediate Arg-dependent regulation, we expressed a nanoLUC luciferase (nanoLUC) reporter enzyme from the *Tg*ApiAT1 5’ region in a strain that expressed a firefly luciferase (fLUC) reporter from the α-tubulin 5’ region (**Fig 2B**). We cultured this ‘dual reporter’ strain at [Arg] ranging from 10 μM to 5 mM for 40–42 hr and measured nanoLUC- and fLUC-dependent luminescence. NanoLUC-dependent luminescence decreased with increasing [Arg], whereas fLUC-dependent luminescence remained unchanged (**S2 Fig**). This enabled fLUC luminescence to be used as a normalising factor, with the nanoLUC:fLUC luminescence ratio providing a measure of Arg-dependent regulation mediated by the 5’ region of the gene encoding *Tg*ApiAT1. There was a significant decrease in the nanoLUC:fLUC ratio as [Arg] increased, with a 55-fold decrease in parasites grown at 5 mM Arg relative to parasites grown at 10 μM Arg (**Fig 2B**). Expression of nanoLUC from the α-tubulin 5’ region revealed no Arg-dependent regulation (**S2 Fig**), ruling out the possibility that nanoLUC expression is itself Arg-dependent. We conclude that the 5’ region of the gene encoding *Tg*ApiAT1 is both *necessary* and *sufficient* to mediate Arg-dependent regulation of the *Tg*ApiAT1 protein.

### *Tg*ApiAT1 abundance is regulated by the availability of other nutrients, including lysine, in an opposite manner to arginine

**Fig 3:**
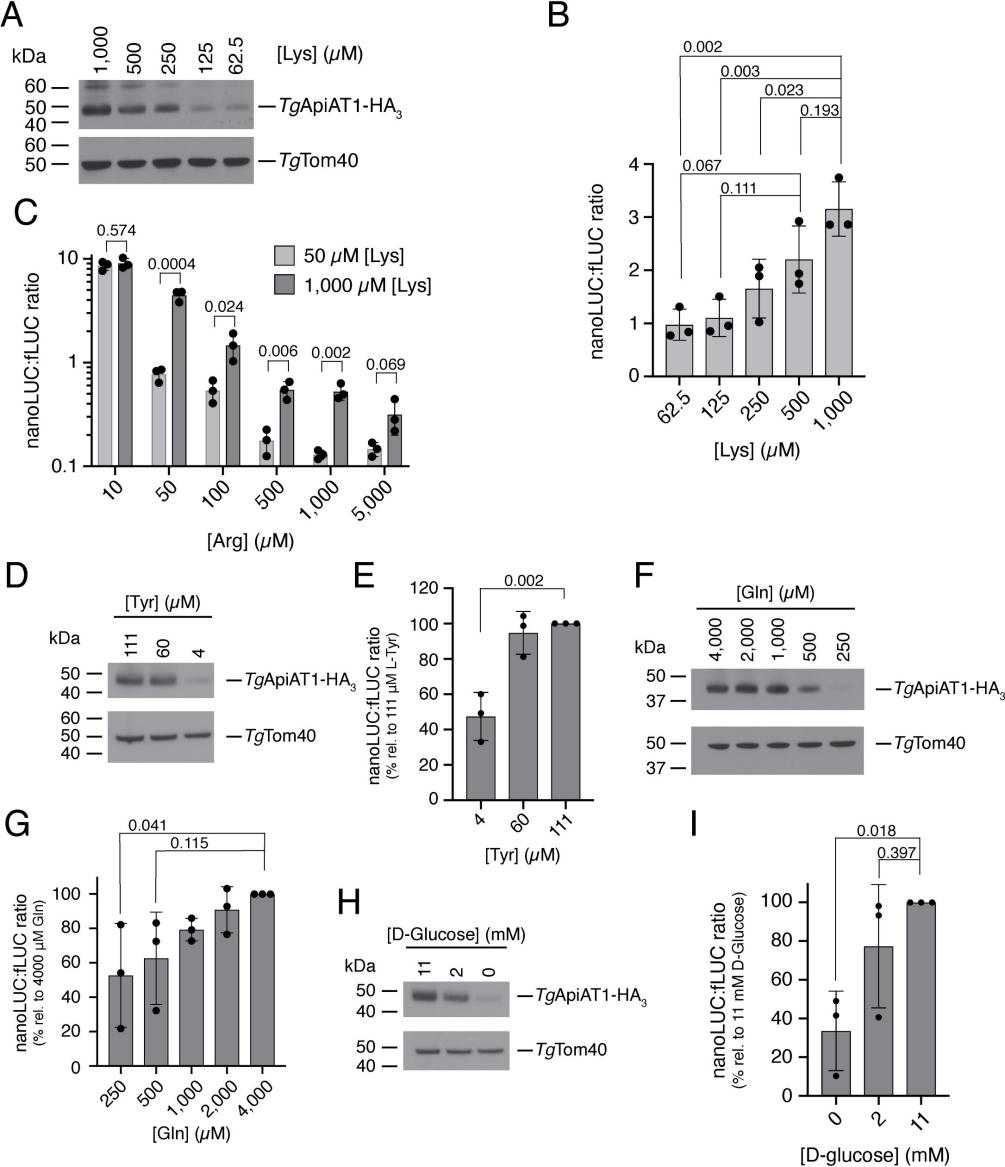
*Tg*ApiAT1 regulation is mediated by a range of nutrients. (**A**, **D**, **F**, **H**) Western blots of *Tg*ApiAT1-HA_3_ in parasites grown at a range of (**A**) [Lys], (**D**) [Tyr], (**F**) [Gln], or (**H**) [D-glucose] in the growth medium. *Tg*Tom40 is a loading control. Data are representative of three independent experiments. (**B**) nanoLUC:fLUC ratio in parasites grown in media containing a range of concentrations of Lys. Data represent the mean ± SD from three independent experiments. *P* values were calculated using a one-way ANOVA with Tukey’s multiple comparisons test. *P* values not shown were > 0.40. (**C**) nanoLUC:fLUC ratios in parasites grown in media containing a range of [Arg] and either 50 μM Lys (light grey) or 1 mM Lys (dark grey). Data represent the mean ± SD from three independent experiments. *P* values were calculated using unpaired t-tests, not assuming equal variance (d.f. = 4). (**E**, **G**, **I)** nanoLUC:fLUC ratios in parasites grown in media containing a range of concentrations of (**E**) Tyr, (**G**) Gln, or (**I**) D-glucose. Data represent the mean ± SD from three independent experiments, with the ratios normalised to the condition with the highest nutrient concentration. *P* values were calculated using a one-way ANOVA with Dunnett’s multiple comparisons test, comparing the normalised nanoLUC:fLUC ratios at each nutrient concentration to the condition containing the highest concentration tested. *P* values not shown were > 0.50.

Next, we asked whether *Tg*ApiAT1 expression is regulated by the availability of other nutrients. Our previous study revealed a connection between the uptake of Arg and Lys in *T*. *gondii* [15]. We therefore measured the abundance of *Tg*ApiAT1-HA_3_ in parasites grown in media containing from 62.5 μM to 1 mM Lys at a constant 50 μM Arg. We observed increased protein abundance with increased [Lys] (**Fig 3A**), the *opposite* effect to what was observed with increasing [Arg]. Similarly, when the dual reporter strain was cultured in media ranging from 62.5 μM to 1 mM Lys and a constant 50 μM Arg for two days, the nanoLUC:fLUC ratio increased with increasing [Lys] (**Figs 3B** and **S3A**). To investigate this further, we measured the nanoLUC:fLUC luminescence ratio at a range of [Arg] at high (1 mM) or low (50 μM) Lys. At all but the lowest Arg concentration tested (*i*.*e*. 10 μM), the nanoLUC:fLUC luminescence ratio measured in parasites grown at high [Lys] was greater than that measured in parasites grown at low [Lys] (**Fig 3C**). Together these results indicate that [Lys] influences *Tg*ApiAT1 expression in the *opposite* manner to [Arg].

We examined the effects of the concentration of a range of other nutrients, including L-tyrosine (Tyr), L-glutamine (Gln) and D-glucose, on the nanoLUC:fLUC luminescence ratio in the dual reporter strain and on *Tg*ApiAT1-HA_3_ protein abundance. At the lowest concentration of each nutrient tested, we observed decreased *Tg*ApiAT1-HA_3_ protein abundance and a significantly decreased nanoLUC:fLUC ratio (**Fig 3D–3I**; *P* < 0.05; **S3B–S3D Fig**). The lowest concentrations tested for Tyr and Gln were close to the minimal amount of those nutrients required for optimal parasite growth [14,18]. This is consistent with the hypothesis that *Tg*ApiAT1 abundance can be negatively regulated through a general amino acid/nutrient starvation response in the parasite [18], and that this regulation is mediated by the 5’ upstream region of *Tg*ApiAT1. These hypotheses were not further investigated here.

**Fig 4:**
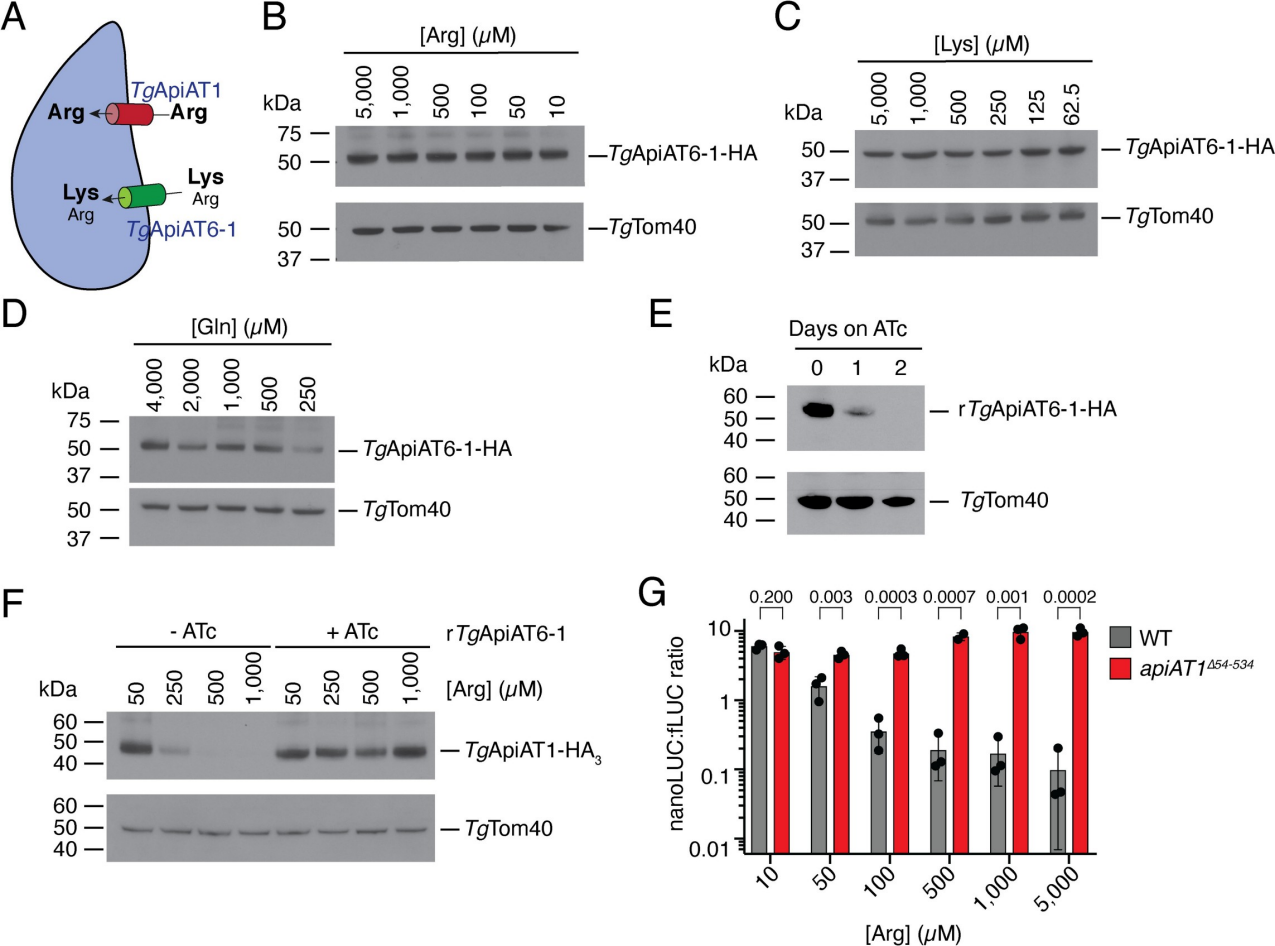
*Tg*ApiAT1 regulation is dependent on transporter-mediated uptake of Arg and Lys into the parasite. (**A**) Model of Arg uptake by *T*. *gondii*. *Tg*ApiAT1 is a selective Arg transporter, while *Tg*ApiAT6-1 is a cationic amino acid transporter with a high affinity for Lys and a lower affinity for Arg. (**B-D**) Western blots measuring the abundance of *Tg*ApiAT6-1-HA-expressing parasites grown at a range of (**B**) [Arg], (**C**) [Lys], or (**D**) [Gln] in the growth medium. *Tg*Tom40 is a loading control. Data are representative of that from two independent experiments. (**E**) Western blot measuring the abundance of r*Tg*ApiAT6-1-HA_3_ upon the addition ATc for 0 to 2 days. *Tg*Tom40 is a loading control. Data are representative of three independent experiments. (**F**) Western blot of *Tg*ApiAT1-HA_3_ in r*Tg*ApiAT6-1 parasites grown in the absence or presence of ATc, and at a range of [Arg] in the growth medium. *Tg*Tom40 is a loading control. Western blots are representative of three independent experiments. (**G**) nanoLUC:fLUC ratios in WT and *apiAT1*^*Δ54–534*^ parasites expressing nanoLUC from the *Tg*ApiAT1 5’ region and fLUC from the α-tubulin 5’ region, and cultured at a range of [Arg]. Data represent the mean ± SD from two or three independent experiments. *P* values were calculated using unpaired t-tests, not assuming equal variance (d.f. = 4). Note that the data from the WT experiments were also included in replicates for the data shown in Fig 2B.

The effect of [Lys] on the expression of *Tg*ApiAT1 was explored further. In a previous study, we demonstrated the presence of a cationic amino acid transporter that has a higher affinity for Lys than for Arg [15]. We have recently identified this protein as being *Tg*ApiAT6-1 (TGME49_240810; [17]), another plasma membrane-localized member of the ApiAT family [14]. *Tg*ApiAT6-1 can take up sufficient Arg for parasite growth in the absence of *Tg*ApiAT1 if the concentration of Lys, a competitive inhibitor of Arg uptake via the transporter, is low (**Fig 4A**; [15,17]). Using an HA-tagged *Tg*ApiAT6-1 strain [14], we asked whether *Tg*ApiAT6-1-HA_3_ abundance is regulated during growth in media containing a range of amino acid concentrations. We found that the abundance of *Tg*ApiAT6-1-HA_3_ did not differ in any of the Arg or Lys concentrations tested (**Fig 4B and 4C**) although, as for *Tg*ApiAT1-HA_3_, we did observe a decrease in protein abundance at low [Gln] (**Fig 4D**), perhaps indicative of multiple transporters being regulated by a general nutrient starvation response.

The data from **Figs 1 and 3** indicate that [Arg] and [Lys] have opposite effects on *Tg*ApiAT1 regulation. We considered two hypotheses to explain these data:

1. That *Tg*ApiAT1 expression is regulated directly by [Lys].
2. That the effect of Lys on *Tg*ApiAT1 expression is a consequence of the effect of Lys on [Arg] within the parasite. Specifically, that the increased [Lys] in the culture medium results in increasing competition by Lys for Arg uptake by the *Tg*ApiAT6-1 transporter, resulting in decreased uptake of [Arg] through *Tg*ApiAT6-1. This results in reduced [Arg] in the parasite, which subsequently results in an increase in *Tg*ApiAT1 expression.

To distinguish between these two possibilities, we utilized a previously-described regulatable *Tg*ApiAT6-1 (r*Tg*ApiAT6-1) parasite strain, in which *Tg*ApiAT6-1 expression can be knocked down through the addition of anhydrotetracycline (ATc), resulting in defects in Arg uptake into the parasite [17]. We introduced a HA tag into the r*Tg*ApiAT6-1 locus and found that *Tg*ApiAT6-1-HA protein was undetectable after two days growth in ATc (**Fig 4E**), consistent with our initial characterisation of this strain [17]. We then introduced a HA tag into the *Tg*ApiAT1 locus of the original r*Tg*ApiAT6-1 strain and grew parasites in the absence or presence of ATc at [Arg] ranging from 50 μM to 1 mM. *Tg*ApiAT1-HA_3_ abundance decreased with increasing [Arg] in the absence of ATc (in which *Tg*ApiAT6-1 is expressed) but remained invariant with varying [Arg] when ATc was added (and *Tg*ApiAT6-1 was depleted; **Fig 4F**). These data, showing that limiting Arg-uptake through *Tg*ApiAT6-1 leads to an increase in *Tg*ApiAT1 expression, are consistent with the second hypothesis–that the Lys-dependent upregulation of *Tg*ApiAT1 **(Fig 3A–3C**) results from reduced [Arg] in the parasite rather than increased [Lys] *per se*.

In a previous study we found that knockout of *Tg*ApiAT1 led to decreased Arg uptake, which is expected to lead to reduced [Arg] in the parasite [15]. To explore further the relationship between parasite [Arg] and *Tg*ApAT1 regulation, we introduced a ‘knockout’ frameshift mutation in the *Tg*ApiAT1 locus of the dual luciferase reporter strain, generating a strain we termed *apiAT1*^*Δ54–534*^. As demonstrated previously for parasites lacking *Tg*ApiAT1 [14,15], *apiAT1*^*Δ54–534*^ parasites exhibited reduced proliferation over an 8-day growth assay in Dulbecco’s modified Eagle’s medium (DME, which contains 400 μM Arg and 800 μM Lys) but grew normally in RPMI (which contains 1.15 mM Arg and 200 μM Lys) (**S4 Fig**). We grew the *apiAT1*^*Δ54–534*^ strain in modified RPMI containing 10 μM to 5 mM Arg for 42 hr and measured the nanoLUC:fLUC luminescence ratio. In contrast to WT parasites, the nanoLUC:fLUC ratio in the *apiAT1*^*Δ54–534*^ strain did not decrease with increasing [Arg] (**Fig 4G**).

Taken together, the data from **Fig 4** indicate that Arg uptake through both *Tg*ApiAT1 and *Tg*ApiAT6-1 modulate the Arg-dependent regulation of *Tg*ApiAT1. The loss of *Tg*ApiAT1, the loss of *Tg*ApiAT6-1, or an increase in [Lys] in the growth medium, are all predicted to result in a depletion of cytosolic [Arg] in the parasite [15]. Our data in **Figs 3 and 4** are therefore consistent with the hypothesis that the parasite is able to sense [Arg] in its cytosol, and respond to changes in cytosolic [Arg] by regulating *Tg*ApiAT1 expression.

### *T*. *gondii* parasites modulate *Tg*ApiAT1 expression *in vivo*

**Fig 5:**
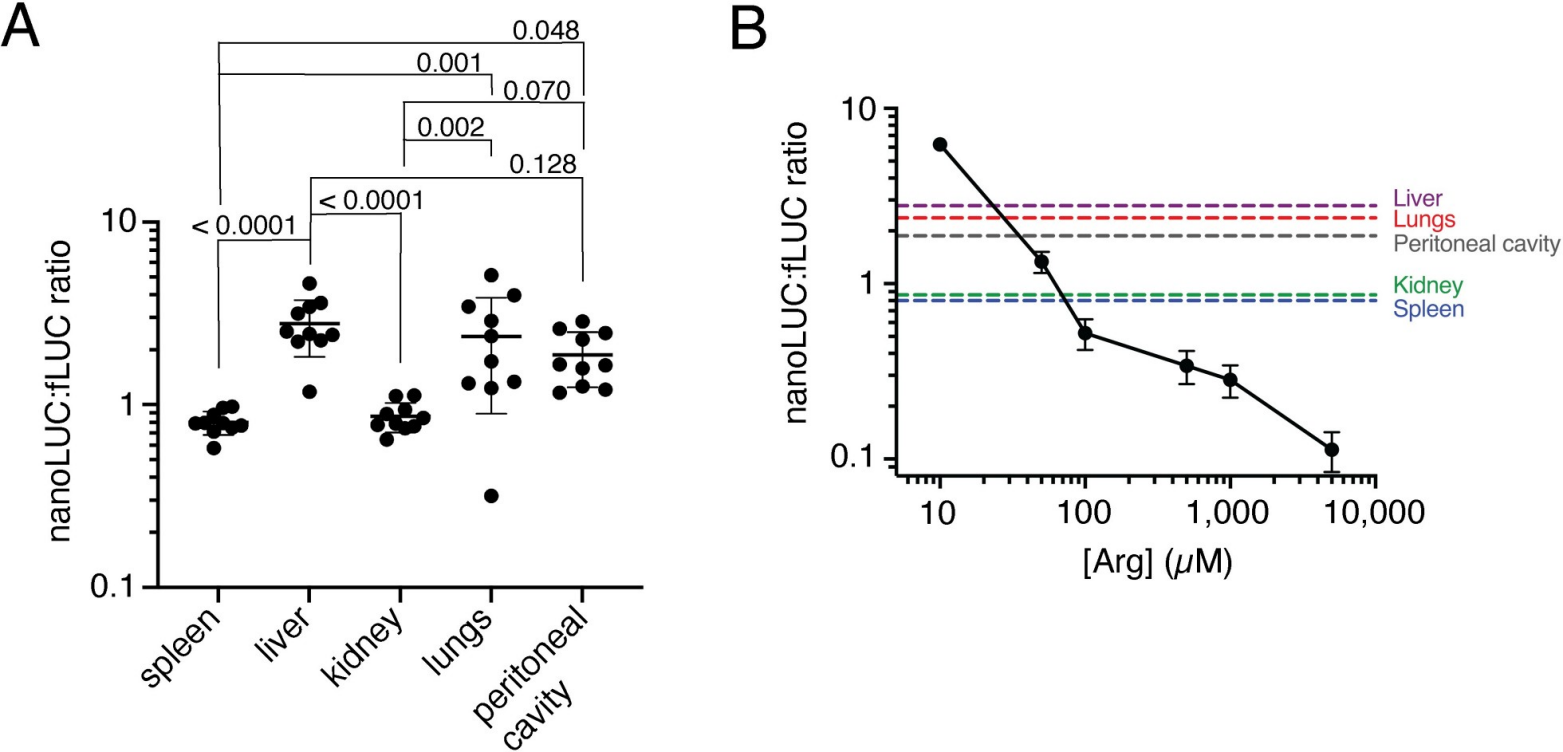
*T*. *gondii* parasites modulate *Tg*ApiAT1 expression *in vivo*. (**A**) NanoLUC:fLUC ratios in *apiAT1 5’*-nanoLUC:*tub 5’-*fLUC dual reporter parasites harvested from a range of organs from infected mice. Mice were infected intraperitoneally with 10^3^ parasites, and euthanised seven days post-infection. Data were derived from two independent experiments with 5 mice each. *P* values were calculated using a one-way ANOVA with Tukey’s multiple comparisons test. *P* values not shown were > 0.60. (**B**) The mean nanoLUC:fLUC luminescence ratios of *apiAT1 5’*-nanoLUC:*tub 5’-*fLUC parasites harvested from various mouse organs and peritoneal cavity in (**A**) shown as dashed lines from the y axis, mapped onto the nanoLUC:fLUC luminescence ratios of *apiAT1 5’*-nanoLUC:*tub 5’-*fLUC parasites grown *in vitro* at a range of [Arg] (solid black line; these data are redrawn from the data depicted in Fig 2B). *In vitro* data represent the mean ± s.e.m. from nine independent experiments.

Our data to this point indicate that *T*. *gondii* parasites are able to sense and respond to changes in [Arg] in their environment. We hypothesise that this enables parasites to modulate Arg uptake through *Tg*ApiAT1 as they encounter different [Arg] during an infection. To investigate whether expression of *Tg*ApiAT1 does vary *in vivo*, we infected mice with dual reporter strain parasites expressing nanoLUC from the wild type *Tg*ApiAT1 5’ region. Seven days after infection, we measured the nanoLUC:fLUC ratio in parasites extracted from a range of organs and from the peritoneal cavity. The ratio varied significantly between organs, with the highest ratios found in the liver, and the lowest in the spleen and kidneys (**Fig 5A**). The different ratios observed in parasites harvested from different organs are consistent with the parasites encountering different [Arg] in these organs during infection. Comparison of the nanoLUC:fLUC luminescence ratios in each organ to those measured in the *in vitro* experiments are consistent with *T*. *gondii* parasites encountering an [Arg] range of ~10–100 μM *in vivo* (**Fig 5B**).

### *Tg*ApiAT1 regulation is mediated by an upstream open reading frame

**Fig 6:**
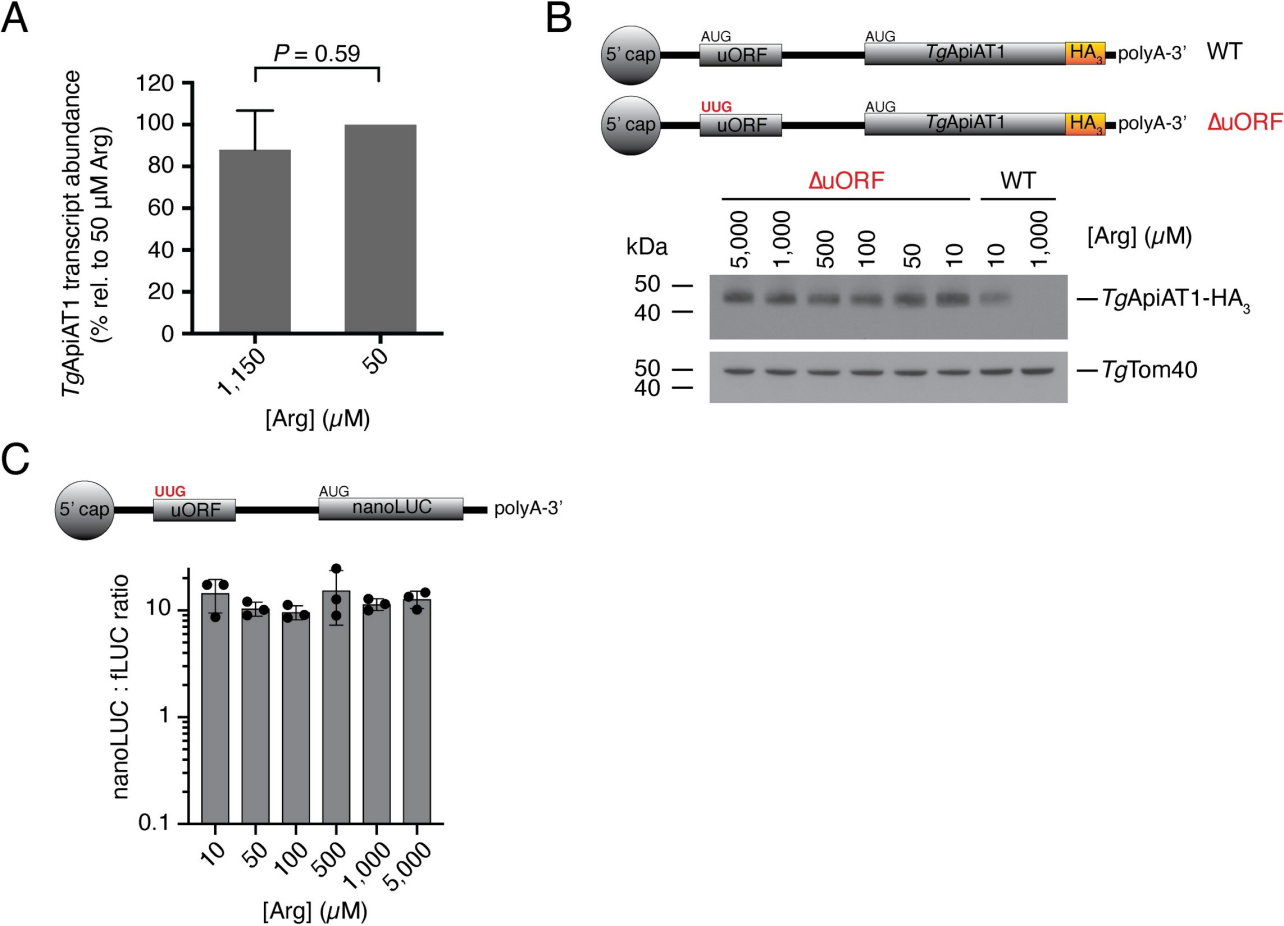
Arg-dependent regulation of *Tg*ApiAT1 occurs post-transcriptionally, and is mediated by an upstream open reading frame. (**A**) Relative *Tg*ApiAT1 transcript abundance in parasites grown at 50 μM and 1.15 mM Arg, normalised to the 50 μM condition. Data represent the mean ± SD from three independent experiments, and the *P* value was calculated using a Student’s t-test. (**B**) Western blot of ΔuORF *Tg*ApiAT1-HA_3_ parasites grown at a range of [Arg] in the growth medium, and probed with anti-HA antibodies. Western blots of WT *Tg*ApiAT1-HA_3_ parasites cultured in 10 μM or 1 mM Arg are included for comparison. *Tg*Tom40 is a loading control. Data are representative of three independent experiments. (**C**) nanoLUC:fLUC ratio in a parasite strain expressing nanoLUC from the *Tg*ApiAT1 5’ region that lacks the uORF start codon (ΔuORF) and fLUC from the α-tubulin 5’ region, and grown at a range of [Arg]. Data represent the mean ± SD from three independent experiments, and were analysed using a one-way ANOVA with Tukey’s multiple comparisons test. All calculated *P* values were 0.55 or greater (not shown).

Finally, we investigated the mechanism by which the 5’ region of the *Tg*ApiAT1 gene regulates *Tg*ApiAT1 expression in response to varying [Arg]. The most common mechanism of 5’-mediated gene regulation in eukaryotes is through regulating transcript abundance [19]. Quantitative real time PCR measurements of *Tg*ApiAT1 transcript abundance in parasites grown at 50 μM compared to 1.15 mM Arg revealed no significant differences (**Fig 6A**), indicating that the Arg-dependent regulation of *Tg*ApiAT1 expression occurs post-transcriptionally.

Post-transcriptional regulation can be mediated by upstream open reading frames (uORFs) in the 5’ untranslated region (5’ UTR) of transcripts [20]. We examined the *Tg*ApiAT1 5’ UTR for start codons of potential uORFs, and identified four. The predicted peptide sequence downstream of one of these candidate start codons is conserved in the related coccidian parasites *Neospora caninum* and *Sarcocystis neurona* (see below). To test whether the conserved candidate uORF has a role in *Tg*ApiAT1 regulation, we used a CRISPR/Cas9 genome editing strategy to convert the ATG start codon of this candidate uORF to TTG in *Tg*ApiAT1-HA_3_-expressing parasites, thus ensuring that the encoded candidate uORF could not be translated. This generated a parasite strain we termed ΔuORF (**Fig 6B**). When this strain was exposed to varying [Arg] there was no Arg-dependent regulation of *Tg*ApiAT1-HA_3_ protein levels (**Fig 6B**), implicating the putative uORF in the Arg-dependent response. We also generated a dual reporter strain in which nanoLUC was expressed from the 5’ region of *Tg*ApiAT1 lacking the putative uORF ATG (**Fig 6C**). We measured the nanoLUC:fLUC luminescence ratio in these parasites grown at a range of [Arg]. Again, we observed no Arg-dependent regulation of expression from the 5’ region of *Tg*ApiAT1 (**Fig 6C**). Together, these data indicate that Arg-dependent regulation of *Tg*ApiAT1 is uORF-mediated.

**Fig 7:**
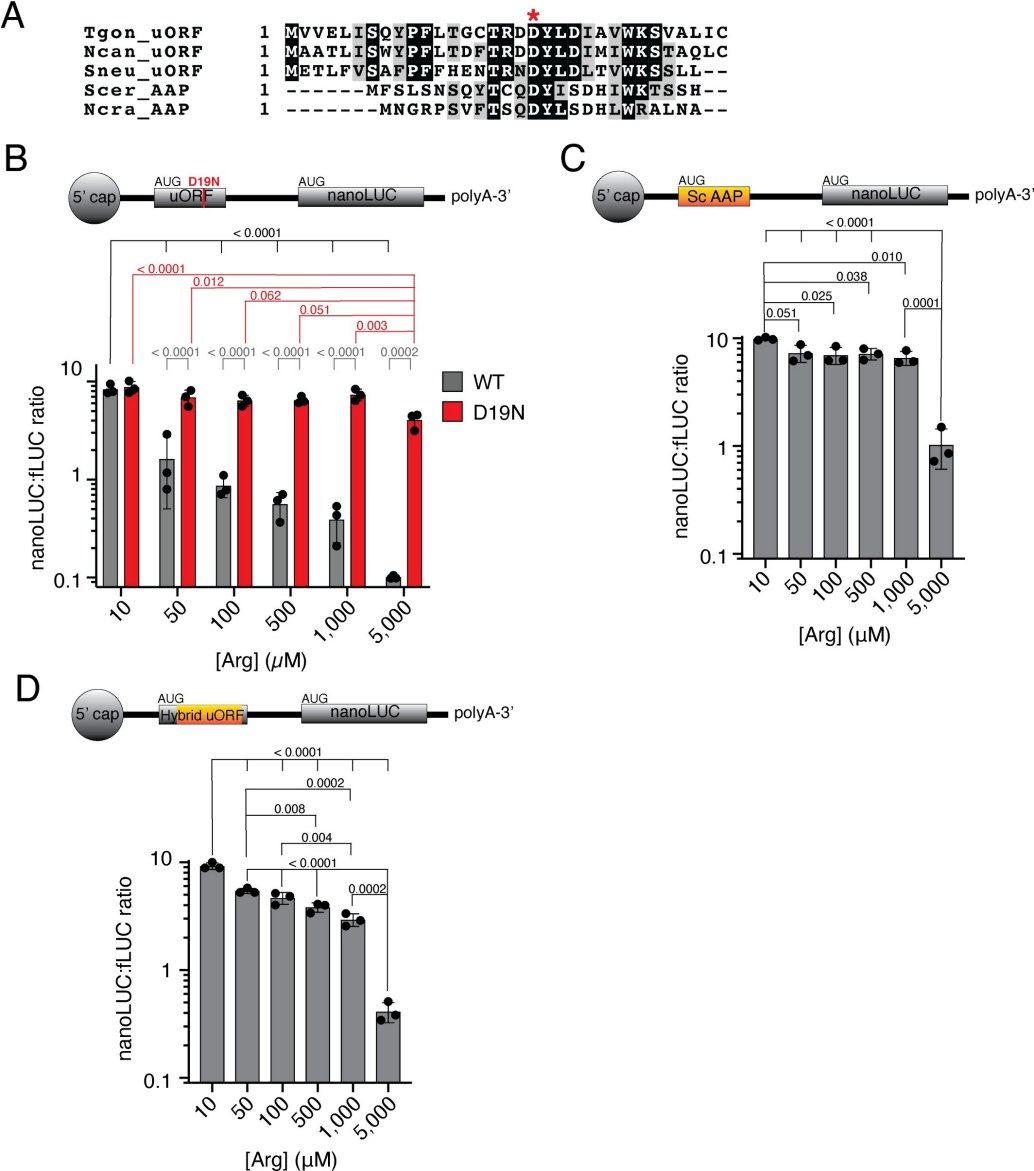
The *Tg*ApiAT1 uORF resembles the Arginine Attenuator Peptide of fungi, and mediates regulation of *Tg*ApiAT1 in a peptide sequence-dependent manner. (**A**) Multiple sequence alignment of the uORF-encoded peptide sequences of ApiAT1 homologues in *T*. *gondii* (Tgon_uORF) and the related coccidian parasites *Neospora caninum* (Ncan_uORF) and *Sarcocystis neurona* (Sneu_uORF), and the arginine attenuator peptides of the fungi *Saccharomyces cerevisiae* (Scer_AAP) and *Neurospora crassa* (Ncra_AAP). The conserved aspartate residue at position 19 of the *Tg*ApiAT1 uORF is highlighted with an asterisk. (**B**) NanoLUC:fLUC ratios in WT and *Tg*ApiAT1^uORF D19N^ (D19N) parasites grown at a range of [Arg]. Data represent the mean ± SD from three independent experiments. *P* values were calculated using a one-way ANOVA with Tukey’s multiple comparisons test. *P* values not shown were > 0.20. (**C-D**) NanoLUC:fLUC ratios in (**C**) *Tg*ApiAT1^ScAAP^ or (**D**) *Tg*ApiAT1^hybrid uORF^ parasites grown at a range of [Arg]. Data represent the mean ± SD from three independent experiments. *P* values were calculated using a one-way ANOVA with Tukey’s multiple comparisons test. *P* values not shown were > 0.20.

uORFs can regulate protein translation in a range of ways, including, in a few instances, through the peptide encoded by the uORF [20,21]. The peptide sequence encoded by the *Tg*ApiAT1 uORF peptide sequence is conserved in closely related coccidian parasites such as *N*. *caninum* and *S*. *neurona* (**Fig 7A**). To test whether the peptide sequence of the *Tg*ApiAT1 uORF is important for regulating translation of the downstream main ORF, we mutated the conserved aspartate residue at position 19 of the *Tg*ApiAT1 uORF to asparagine (D19N; a mutation mediated by a single base pair change in the transcript; **Fig 7A**) and used the mutated *Tg*ApiAT1 5’ UTR to drive nanoLUC expression in a dual reporter strain. We grew D19N parasites in media containing a range of [Arg] and measured nanoLUC:fLUC luminescence ratios. In contrast to a control strain expressing nanoLUC from the WT *Tg*ApiAT1 5’ region, the nanoLUC:fLUC ratio in D19N parasites did not decrease with increasing [Arg] at most concentrations tested, although we observed a slight but significant reduction in the nanoLUC:fLUC ratio at 5 mM (**Fig 7B**). Expression from the *Tg*ApiAT1 5’ UTR was, therefore, largely unresponsive to variations in [Arg] in D19N parasites, consistent with the hypothesis that the peptide sequence of the *Tg*ApiAT1 uORF is important for Arg-dependent regulation.

The best characterised example of uORF peptide-mediated regulation in the literature is the so-called arginine attenuator peptide (AAP) of fungi [22]. This peptide is encoded by the uORF of the gene encoding carbamoyl phosphate synthetase (Arg2), an arginine biosynthesis enzyme. Like the uORF peptide of *Tg*ApiAT1, the AAP is responsive to Arg, and mediates repression of the downstream open reading frame under arginine-replete conditions [23]. The *Tg*ApiAT1 uORF peptide has some sequence similarity to the AAP from *Saccharomyces cerevisiae* and *Neurospora crassa*, including in the conserved aspartate that is critical for both *Tg*ApiAT1 uORF and AAP function (**Fig 7A and 7B**; [22]). To test whether the *S*. *cerevisiae* AAP (*Sc*AAP) can replace the function of the *Tg*ApiAT1 uORF peptide, we expressed nanoLUC from a modified *Tg*ApiAT1 5’ region in which the native uORF was replaced by a uORF encoding *Sc*AAP in a dual reporter strain. We grew the resultant strain at a range of [Arg] and measured the nanoLUC:fLUC ratio. We observed a small but significant decrease in the nanoLUC:fLUC ratio with increasing [Arg], most noticeably at the highest [Arg] tested (**Fig 7C**).

The *Tg*ApiAT1 uORF peptide (33 amino acids) is larger than *Sc*AAP (25 amino acids). We generated a ‘hybrid’ uORF that encoded the first seven and last two amino acids of the *Tg*ApiAT1 uORF either side of the *Sc*AAP, generating a peptide of the same length as the *Tg*ApiAT1 uORF, and incorporated this into the *Tg*ApiAT1 5’ region driving nanoLUC in a dual reporter strain. We measured the nanoLUC:fLUC ratio at a range of [Arg] and observed a significant decrease in the ratio with increased [Arg] (**Fig 7D**). Together, these data indicate that *Sc*AAP can partially complement the function of the *Tg*ApiAT1 uORF in mediating Arg-dependent regulation in *T*. *gondii*, suggesting that similar mechanisms of peptide sequence-dependent regulation may be occurring.

## Discussion

This paper describes what is, to our knowledge, the first example of substrate-mediated regulation of a transporter in apicomplexan parasites. It adds to a growing body of literature on the ability of apicomplexan parasites to sense and respond to changes in nutrient availability [7–10,24]. Our data indicate that *T*. *gondii* parasites can sense [Arg] in their environment, and respond by regulating the abundance of the Arg transporter *Tg*ApiAT1. The ability of the parasite to regulate *Tg*ApiAT1 abundance may contribute to enabling the parasite to take up sufficient Arg to facilitate its proliferation as it encounters variable [Arg] across the course of an infection, and may play a role in the ability of *T*. *gondii* to infect a broad range of cell types in different hosts.

Arg uptake into *T*. *gondii* is mediated by the combined action of *Tg*ApiAT1, a selective Arg transporter, and *Tg*ApiAT6-1, a broad cationic amino acid transporter that is particularly important for Lys uptake into the parasite [15,17]. *Tg*ApiAT6-1 is constitutively expressed, regardless of the cationic amino acid concentrations that the parasite encounters (**Fig 4**), whereas *Tg*ApiAT1 abundance is influenced in an antagonistic manner by the concentrations of Arg and Lys in the growth medium (**Figs 1** and **3**), and is expressed at different levels in different organs during infection (**Fig 5**). We hypothesise that *Tg*ApiAT1 regulation enables *T*. *gondii* parasites to respond to different [Arg] that parasites encounter in different *in vivo* environments. When the [Arg] that parasites encounter in their *in vivo* environment is low, reduced Arg uptake through *Tg*ApiAT6-1 leads to decreased [Arg] in the parasite, and promotes an upregulation of *Tg*ApiAT1 expression. *Tg*ApiAT1 regulation enables parasites to modulate Arg uptake from the host cell, thereby exerting tight control over their intracellular [Arg].

Using a novel, Arg-dependent dual luciferase-expressing parasite strain, we demonstrate that parasite expression of *Tg*ApiAT1 varies between different organs during a mouse infection (**Fig 5**). This is consistent with parasites encountering different Arg concentrations in the different organs they infect. We attempted to determine Arg levels in these various organs experimentally, but were unable to obtain reproducible data. Nevertheless, Arg metabolism is known to differ in different organs of mice and other mammals. For example, kidneys are a major site of Arg synthesis [25], whereas liver cells have a high activity of the arginine degrading enzyme arginase [26]. This is reflected in the [Arg] in these organs, with Arg levels in kidneys being higher than in the liver in many mammals, including mice and other rodents [25,27,28]. In concurrence with these studies, we demonstrate that *Tg*ApiAT1 expression is significantly higher in the liver than in the kidneys (**Fig 5**). In addition to a likely role for Arg in modulating *Tg*ApiAT1 expression *in vivo*, our *in vitro* data indicate that Lys and other nutrients also contribute to *Tg*ApiAT1 regulation, and it is likely that the abundances of these nutrients contribute to *Tg*ApiAT1 regulation *in vivo*. Together, our data provide experimental evidence that parasites are encountering different nutrient conditions in different physiological niches in their hosts, and that they are able to sense and respond to these differences.

As well as encountering different [Arg] in different organs, parasites may also encounter different [Arg] across the course of an infection. Infection by *T*. *gondii* parasites can lead to an upregulation of host enzymes that utilize Arg as a substrate, including arginase and nitric oxide synthases [29–31]. Secreted parasite proteins such as *Tg*ROP16 and *Tg*GRA15 promote the upregulation of these enzymes in hosts [11,32], and these effector proteins are important modulators of parasite virulence in some strains of *T*. *gondii* [33,34]. Having established a means of estimating the [Arg] that parasites encounter *in vivo* (**Fig 5**), it will now be of interest to determine whether *Tg*ApiAT1 expression changes across the course of an infection in response to the upregulation of Arg-dependent enzymes in hosts, and the role that parasite effector proteins may have in modulating host [Arg] in some strains of the parasite.

Our study indicates that *Tg*ApiAT1 is regulated by the availability of a range nutrients, including glucose, and the amino acids Gln and Tyr, although the direction of regulation is inverse to the regulation observed in response to changes in [Arg] (with *Tg*ApiAT1 abundance decreasing upon limitation of these nutrients) (**Fig 3**). *Tg*ApiAT1 downregulation was observed at concentrations of Gln and Tyr that were close to, or below, the levels required for optimal parasite growth [14,18]. Thus, in addition to being regulated by an Arg-specific mechanism, *Tg*ApiAT1 abundance may be regulated as part of a more general starvation response. The response of *T*. *gondii* parasites to Gln starvation has some similarities to the GCN2-dependent translational regulation that occurs during the starvation response of mammalian cells [18]. The putative starvation response was observed both when measuring *Tg*ApiAT1 protein abundance, and in a strain in which nanoLUC was driven by the 5’ region of the *Tg*ApiAT1 gene. This is consistent with the starvation response being mediated by the 5’ region of *Tg*ApiAT1. It remains to be determined whether the mechanism mediating this general starvation response is similar to the specific Arg-dependent response.

Regulation of cationic amino acid transporters in response to the availability of their substrates is observed in a range of organisms, including mammals and the protozoan parasite *Leishmania donovani* [35,36]. A recent study by Augusto and colleagues demonstrated that *T*. *gondii*-mediated depletion of [Arg] in mammalian host cells resulted in increased abundance of the CAT1 cationic amino acid transporter of mammalian host cells [37]. Our data indicate an additional layer of complexity in mediating Arg acquisition by the parasite, with parasites able to modulate the amount of Arg that they take up from their host by regulating the level of expression of their primary Arg uptake transporter.

*L*. *donovani* contains a single Arg transporter (*Ld*AAP3) that is unrelated to *Tg*ApiAT1, and which is upregulated in response to a reduction of [Arg] in the growth medium [35]. Arg-dependent changes in *Ld*AAP3 protein levels result from changes to *Ld*AAP3 transcript abundance [35], and so is mechanistically different from the uORF-based mechanism that regulates *Tg*ApiAT1 expression in *T*. *gondii*. *L*. *donovani* is an intracellular parasite for part of its life cycle, inhabiting macrophages of its mammalian hosts. The presence of regulatory mechanisms in Arg transporters from two unrelated intracellular parasites suggests that tight regulation of Arg uptake may be important for the survival of intracellular pathogens in their host.

We demonstrated that Arg-dependent regulation of *Tg*ApiAT1 is mediated by a uORF in the *Tg*ApiAT transcript (**Figs 6 and 7**). uORFs appear to be abundant in *T*. *gondii* transcripts [38], and the uORF of *Tg*ApiAT1 represents the first characterised example of a functional uORF in these parasites [39]. Our data indicate that the peptide encoded by the uORF plays a role in the Arg-dependent regulation of *Tg*ApiAT1 expression (**Fig 7**). This is one of only a few known cases in which the peptide of a uORF appears to be critical for regulating translation of the downstream main ORF [20]. How the *Tg*ApiAT1 uORF mediates Arg-dependent *Tg*ApiAT1 regulation requires further experimentation. The best studied example of peptide-dependent uORF regulation is the AAP of fungi, which regulates the Arg-dependent translation of the arginine biosynthesis enzyme Arg2 [40]. The AAP mediates ribosome stalling on the Arg2 transcript in Arg-replete conditions, possibly by blocking the ribosome exit tunnel in an Arg-dependent manner [23,41,42]. The sequence of the *Tg*ApiAT1 uORF resembles that of the AAP (**Fig 7A**), with one of the conserved residues being critical for *Tg*ApiAT1 uORF function, and the yeast AAP being partially functional in *T*. *gondii* (**Fig 7B–7D**). This suggests that the *Tg*ApiAT1 uORF may function in a similar manner to the AAP. Given that *T*. *gondii* and fungi are separated by ~1.5 billion years of evolution, and that the *Tg*ApiAT1 uORF peptide appears restricted to *T*. *gondii* and its closest relatives, a conserved function between these uORFs would represent a remarkable example of convergent evolution.

## Methods

### Ethics statement

All animal research was conducted in accordance with the National Health and Medical Research Council’s Australian Code for the Care and Use of Animals for Scientific Purposes, and the Australian Capital Territory Animal Welfare Act 1992. Mice were maintained and handled in accordance with protocols approved by the Australian National University Animal Experimentation Ethics Committee (protocol number A2016/42).

### Parasite culture

Parasite cultures were maintained in human foreskin fibroblasts (a kind gift from Holger Schlüter, Peter MacCallum Cancer Centre) in a humidified 37°C incubator at 5% CO_2_. Host cells were checked periodically for *Mycoplasma* infection. Unless otherwise indicated in the text, parasites were cultured in RPMI supplemented with 1% (v/v) foetal calf serum, 200 μM glutamine, 50 U/ml penicillin, 50 μg/ml streptomycin, 10 μg/ml gentamicin, and 0.25 μg/ml amphotericin b, as described [15]. For all ‘homemade’ media in which we varied the concentrations of nutrients, we used 1% (v/v) dialysed foetal calf serum. Unless otherwise indicated in the manuscript, parasites were cultured in particular media conditions for two days before harvesting for subsequent assays. Where applicable, ATc was added to a final concentration of 0.5 μg/ml. Experiments to measure the effects of a range of [Arg] on *Tg*ApiAT1-HA_3_ abundance or *apiAT1* 5’-nanoLUC activity were performed in RPMI containing 200 μM Lys, unless otherwise indicated. Experiments to measure the effects of [Lys], [Tyr], [Gln] and [D-glucose] on *Tg*ApiAT1 regulation were performed in medium containing 50 μM Arg unless otherwise indicated. Plaque assays were performed in 25 cm^2^ tissue culture flasks, with 500 parasites added to a flask. Parasites were grown for 8 days before being stained in a solution of 2% (w/v) crystal violet, 20% (w/v) ethanol and 0.8% (w/v) ammonium acetate. To induce bradyzoite formation, 1.4 × 10^6^ tachyzoites were inoculated into a 25 cm^2^ tissue culture flask with confluent human foreskin fibroblasts and allowed to proliferate for 20 hr in standard growth medium. The growth medium was replaced with alkaline RPMI supplemented with 25 mM HEPES (pH 8.2–8.4), and the infected host cells were then cultured for a further six days at ambient CO_2_ levels. Intracellular bradyzoites were mechanically egressed from host cells using a 26 gauge needle, then further disrupted using a 30 gauge needle before sample preparation for SDS-PAGE.

### Mouse infections

Freshly egressed, dual reporter strain parasites were filtered through a 3 μm polycarbonate filter, washed once in phosphate-buffered saline (PBS), and resuspended to 1 × 10^4^ parasites/ml in PBS. 6–8 week-old, female Balb/c mice were inoculated intraperitoneally with 1 × 10^3^ parasites using a 26-gauge needle. Mice were weighed regularly and monitored for symptoms of toxoplasmosis (weight loss, ruffled fur, lethargy and hunched posture). At day 6, mice were imaged using an IVIS imaging system to confirm infection, as described [43]. Briefly, mice were injected intraperitoneally with 200 μl of 15 mg/ml D-luciferin in PBS, anaesthetised with 2.5% isofluorane in oxygen in an anaesthetic chamber using an XGI-8 anaesthesia system, and imaging was performed on an IVIS Spectrum imaging system 10 min post-injection. Anaesthesia was maintained during imaging by application of 2.5% isofluorane in oxygen via a nose cone. All mice were euthanised at day 7 of the experiment and dissected to remove organs for dual luciferase assay measurements, as described below. We also euthanised and analysed two uninfected mice to determine background luminescence levels found in each tested organ.

### Generation of genetically modified *T*. *gondii* strains

The *Tg*ApiAT1 gene encodes a 3,548 bp transcript containing a 703 bp 5’ UTR and a 1,040 bp 3’UTR, with a single intron of 203 bp that is spliced to yield the mRNA (**S5 Fig**; www.toxodb.org; [44]). The main ORF encodes for a protein of 534 amino acids. The uORF that participates in Arg-dependent regulation of the downstream main ORF is 99 bp in length and occurs between base pairs 284 and 382 of the 5’ UTR (**S5 Fig**). To incorporate a 3xHA tag into the *Tg*ApiAT1 genomic locus, we adopted a CRISPR/Cas9 genome editing strategy. We introduced a single guide RNA (gRNA) targeting the 3’ region of the *Tg*ApiAT1 locus into the vector pSAG1::Cas9-U6::sgUPRT (Addgene plasmid # 54467; [45]) using Q5 site-directed mutagenesis (New England Biolabs) with the primers ApiAT1 3’ gRNA fwd and generic rvs (**S2 Table**), as described previously [45]. We generated a donor DNA containing the 3xHA-tag flanked by sequence homologous to the *Tg*ApiAT1 locus either side of the *Tg*ApiAT1 stop codon as a gBlock (IDT; **S2 Table**). We amplified the ‘*Tg*ApiAT1-HA_3_’ gBlock DNA (IDT) by polymerase chain reaction (PCR) using the primers ApiAT1 3’ edit fwd and rvs (**S2 Table**). We co-transfected the gRNA/Cas9-GFP-expressing vector and the donor DNA into TATiΔ*ku80* [46], PrugniaudΔ*ku80Δhxgprt*/*ldh2*-GFP [47], or r*Tg*ApiAT6-1 strain parasites, and sorted GFP-expressing clones 2–3 days post-transfection, as described [14,48].

To generate the dual luciferase reporter strain, we first generated a strain that expressed firefly luciferase (fLUC) under the control of the *T*. *gondii* α-tubulin 5’ region. We digested the vector pTub8-rsLUC (a kind gift from Boris Striepen, U. Penn) with *Spe*I and *Not*I and ligated this into the equivalent sites of pDTG [49], a vector that encodes a pyrimethamine-resistance marker. We transfected this plasmid into RHΔ*hxgprt* [50] strain parasites, selected on pyrimethamine, and obtained clonal parasites by limiting dilution. This generated a strain that we termed the αtub 5’-fLUC strain, which constitutively expressed fLUC from the α-tubulin 5’ region. We next set about generating a plasmid that expressed nanoLUC from the *Tg*ApiAT1 5’ region. First, we generated a vector expressing firefly luciferase (fLUC) under the control of the *Tg*ApiAT1 5’ region. We amplified fLUC with the primers fLUC fwd and fLUC rvs (**S2 Table**) using the LT-3 plasmid [51] (a kind gift from Alex Maier, ANU) as template. We digested the resulting product with *Bgl*II and *Avr*II and ligated this into the equivalent sites of the vector pUgCTH_3_ [15], generating a vector we termed pUgCT-fLUC-HA_3_. We PCR amplified the 1.2 kb region upstream of the *Tg*ApiAT1 5’ UTR (*i*.*e*. upstream of the transcript start site) using the primers ApiAT1 5’ fwd and rvs (**S2 Table**), digested the product with *Spe*I and *Asi*SI and ligated into the equivalent sites of pUgCT-fLUC-HA_3_. We then amplified the 5’ UTR of the *Tg*ApiAT1 gene using the ApiAT1 5’ UTR fwd and rvs primers (**S2 Table**), digested the resulting product with *Sbf*I and *Asi*SI, and ligated this into the equivalent sites of the pUgCT-fLUC-HA_3_ vector, terming the resultant vector pUgC-apiAT1 5’-fLUC-HA_3_. Next, we amplified nanoLUC using the nanoLUC fwd and rvs primers (**S2 Table**) and the plasmid pTubNluc-AID-2xHA-DHFR (a kind gift from Boris Striepen, U. Penn) as template. We digested the resulting product with *Asi*SI and *Avr*II, and ligated this into the equivalent site of pUgC-apiAT1 5’-fLUC-HA_3_, generating a vector we termed pUgC-apiAT1 5’-nanoLUC-HA_3_. The resultant plasmid encodes nanoLUC under control of the *Tg*ApiAT1 5’ region. We transfected this plasmid into the αtub 5’-fLUC parasite strain, selected on chloramphenicol, and obtained clonal parasites by limiting dilution. We termed the resultant strain the ‘dual reporter strain’.

Generation of the ATc-regulatable *Tg*ApiAT6-1 and HA-tagged ATc regulatable *Tg*ApiAT6-1-HA_3_ strains were described previously [17].

To generate a frameshifted ‘knockout’ mutation in the *Tg*ApiAT1 locus of the dual reporter strain, we transfected this with a plasmid expressing a gRNA targeting the *Tg*ApiAT1 locus, sorted and cloned parasites 3 days after transfection, and verified that a successful frameshift mutation (a single base pair insertion) had occurred by sequencing the *Tg*ApiAT1 locus, all as described previously [14].

To generate a *Tg*ApiAT1-HA_3_-expressing strain wherein the ATG start codon of the *Tg*ApiAT1 uORF was mutated to TTG, we adopted a CRISPR/Cas9 genome editing strategy. First, we introduced a gRNA targeting the genomic locus that encoded the *Tg*ApiAT1 5’ UTR near the uORF start codon into pSAG1::Cas9-U6::sgUPRT vector using Q5 site-directed mutagenesis with the primers ApiAT1 uORF gRNA fwd and generic rvs (**S2 Table**) as described previously [45]. We generated a donor DNA wherein the ATG of the uORF was mutated to TTG by annealing the complementary primers ApiAT1 ΔuORF fwd and rvs (**S2 Table**), and co-transfected this with the gRNA-expressing vector into *Tg*ApiAT1-HA_3_ strain parasites. We sorted GFP-expressing clones 3 days post-transfection, then sequenced clones to verify successful mutation. In addition to the ATG start codon of the uORF being mutated to TTG, the clone that we characterised had an additional G to C mutation in the protospacer adjacent motif (PAM) site of the gRNA target (13 bp upstream of the ATG codon) designed to prevent gRNA-mediated Cas9 cutting of the chromosome following genome modification, and an unintended G to A mutation 6 bp upstream of the start codon, likely introduced by a mutation in the donor DNA.

To generate a strain expressing nanoLUC from the *Tg*ApiAT1 5’ region in which the uORF ATG start codon was mutated to TTG, we amplified the 5’UTR of the *Tg*ApiAT1 using the ApiAT1 5’ UTR fwd and rvs primers (**S2 Table**), and a ‘*Tg*ApiAT1/ΔuORF 5’UTR’ gBlock (IDT) encoding an altered *Tg*ApiAT1 5’ UTR region in which the start codon of the *Tg*ApiAT1 uORF was mutated to TTG (**S2 Table**). We digested the resultant PCR product with *Pst*I and *Asi*SI, and ligated into the *Sbf*I and *Asi*SI sites of pUgC-apiAT1 5’-nanoLUC-HA_3_. We transfected this vector into the αtub 5’-fLUC strain, selected on chloramphenicol, and obtained clonal parasites by limiting dilution. To generate a strain expressing nanoLUC from the *Tg*ApiAT1 5’ region wherein the native *Tg*ApiAT1 uORF was replaced with the *S*. *cerevisiae* AAP uORF, we amplified a modified *Tg*ApiAT1 5’UTR containing the *S*. *cerevisiae* AAP uORF using the ApiAT1 5’ UTR fwd and rvs primers (**S2 Table**) and a ‘*Tg*ApiAT1/ScAAP uORF 5’UTR’ gBlock (IDT; **S2 Table**). We digested the resultant PCR product with *Pst*I and *Asi*SI, and ligated into the *Sbf*I and *Asi*SI sites of pUgC-apiAT1 5’-nanoLUC-HA_3_. We transfected this vector into the αtub 5’-fLUC strain, selected on chloramphenicol, and obtained clonal parasites by limiting dilution. To generate a strain expressing nanoLUC from the *Tg*ApiAT1 5’ region containing a ‘hybrid’ uORF consisting of the *S*. *cerevisiae* AAP flanked by the 5’ and 3’ regions of the *Tg*ApiAT1 uORF, we amplified a modified *Tg*ApiAT1 5’UTR containing the hybrid *Tg*ApiAT1 uORF using the ApiAT1 5’ UTR fwd and rvs primers (**S2 Table**) and a ‘*Tg*ApiAT1/hybrid uORF 5’UTR’ gBlock (IDT; **S2 Table**). We digested the resultant PCR product with *Pst*I and *Asi*SI, and ligated into the *Sbf*I and *Asi*SI sites of pUgC-apiAT1 5’-nanoLUC-HA_3_. We transfected this vector into the αtub 5’-fLUC strain, selected on chloramphenicol, and obtained clonal parasites by limiting dilution.

To generate a strain expressing nanoLUC from the *Tg*ApiAT1 5’ region wherein the aspartate residue at position 19 of the uORF peptide was mutated to asparagine (D19N), we used a Q5 mutagenesis approach. We followed the manufacturer’s instructions (New England Biolabs), using the pUgC-apiAT1 5’-nanoLUC-HA_3_ plasmid as template, and the uORF D19 fwd and rvs primers (**S2 Table**). We transfected the resultant vector into tub 5’-fLUC strain parasites, selected on chloramphenicol, and obtained clonal parasites by limiting dilution.

To generate a strain that expressed nanoLUC from the α-tubulin 5’ region, we amplified the α-tubulin 5’ region with the primers Tub 5’ fwd and rvs (**S2 Table**), digested the product with *Spe*I and *Asi*SI and ligated into the equivalent sites of the pUgC-apiAT1 5’-nanoLUC-HA_3_, generating a vector we termed pUgC-tub 5’-nanoLUC-HA_3_. We transfected this plasmid into RHΔ*hxpgrt* strain parasites, selected on chloramphenicol, and obtained clonal parasites by limiting dilution.

### Quantitative real time PCR

TATiΔ*ku80* strain parasites were cultured for 2 days in modified RPMI medium containing 50 μM or 1.15 mM Arg. Parasites were mechanically egressed from host cells using a 26 gauge needle, then total RNA was extracted using the Isolate II RNA mini extraction kit (Bioline), according to the ‘cultured cells and tissue’ protocol in the manufacturer’s instructions. cDNA synthesis was performed using the High-Capacity cDNA reverse transcriptase kit (Applied Biosystems) with a random primer mix and 2 μg total RNA from each sample, according to the manufacturer’s instructions. Quantitative real time PCR was performed using a LightCycler 480 system (Roche) with the LightCycler 480 SybrGreen I Master mix, following the manufacturer’s instructions, and using 5 μM primers. The LightCycler 480 conditions were as follows: 10 min preincubation at 95°C, then 45 cycles of 15 sec denaturation (95°C), 15 sec annealing (58°C), and 20 sec elongation (72°C). To detect the abundance of *Tg*ApiAT1 transcript, we used the primers ApiAT1 qrt int fwd and rvs (which amplified *Tg*ApiAT1 cDNA across the intron of the transcript) and ApiAT1 qrt 3’ UTR fwd and rvs (which amplified *Tg*ApiAT1 cDNA from the 3’ UTR of the transcript; **S2 Table**). *Tg*ApiAT1 transcript levels were normalised using α-tubulin (Tub) and glyceraldehyde-3-phosphate dehydrogenase (GAPDH) as housekeeping transcript controls. We amplified these housekeeping controls using the primers Tub qrt fwd and rvs and GAPDH qrt fwd and rvs; **S2 Table**). Raw fluorescence data were exported and analysed using LinRegPCR [52] to perform background subtraction and determine PCR primer efficiency. Samples were then normalized to housekeeping controls using the Pfaffl equation (E_NPT1_^ΔCTNPT1^/E_ref_^ΔCTref^; E = primer efficiency, ΔCT = difference in cycle threshold between samples grown at 1.15 mM and 50 μM Arg, ref = housekeeping controls; [53]) and expressed as percentage relative to that at 50 μM. Three biological replicates were performed and each reaction was done in at least triplicate.

### Western blotting

Protein samples were separated using NuPAGE Bis/Tris gels, as described [15], loading 2.5 × 10^6^ parasite equivalents per lane. Membranes were probed with rat anti-HA antibodies (1:100 to 1:3,000 dilutions; clone 3F10, Sigma, 11867423001), rabbit anti-*Tg*Tom40 [54] (1:2,000 dilution), mouse anti-GFP (1:1,000 dilution; Sigma, 11814460001), mouse anti-BAG1 [55] (1:250 dilution; a kind gift from Louis Weiss, Albert Einstein College of Medicine), rabbit anti-SAG1 (1:1,000 dilution; a kind gift from Michael Panas and John Boothroyd, Stanford University), or mouse anti-*Tg*GRA8 [56] (1:100,000 dilution; a kind gift from Gary Ward, U. Vermont) as primary antibodies, and horseradish peroxidase-conjugated goat anti-rat (1:5,000 to 1:10,000 dilutions; Santa Cruz, sc-2006, or Abcam, ab97057), goat anti-rabbit (1:5,000 to 1:10,000 dilution; Santa Cruz, sc-2004, or Abcam, ab97051), or goat anti-mouse (1:5,000 to 1:10,000 dilution; Santa Cruz, sc-2005) secondary antibodies.

### SWATH-MS proteomic analysis

#### Sample preparation

We undertook a SWATH-MS-based quantitative proteomic approach [16] to establish whether the abundance of proteins changed in parasites grown in media containing low vs high [Arg]. We cultured RHΔ*hxgprt* strain parasites in modified DME containing 50 μM or 1.15 mM Arg, and a constant 800 μM Lys for two days. Our previous data indicate that 50 μM is the minimum [Arg] required for optimal parasite growth [15], and we chose 50 μM as the low [Arg] value (and not a lower concentration) to avoid identifying proteins that change abundance as a result of a general starvation response. We performed five replicates for each condition. Parasites were mechanically egressed through a 26 gauge needle, filtered through a 3 μm polycarbonate filter to remove host cell debris, washed in PBS, then resuspended in a lysis buffer containing 1% (w/v) sodium dodecyl sulfate (SDS), 1 mM dithiothreitol (DTT), 50 mM Tris-HCl, pH 8. SDS was removed by buffer exchange with 100 mM triethylammonium biocarbonate.

#### Sample processing

100 μg of protein from each sample was reduced in 10 mM DTT, alkylated with 20 mM iodoacetamide, then digested by trypsin for 16 hr at 37°C. Digested samples were cleaned up using a detergent removal spin column (Pierce), then dried and resuspended in 100 μL of 2% (v/v) acetonitrile with 0.1% (v/v) formic acid. For one-dimensional information dependent acquisition (1D-IDA), 10 μl of each sample was subjected to nanoLC MS/MS analysis using an Ultra nanoLC (Eksigent) system and Triple TOP 5600 mass spectrometer (AB Sciex). For two-dimensional (2D)-IDA, a pool was prepared from 20 μl of each sample, and separated by high pH reverse phase fractionation on a Agilent 1260 quaternary HPLC system with a Zorbax 300Extend-C18 column, with 12 fractions collected. Each 1D-IDA and 2D-IDA sample was injected onto a Captrap peptide trap (Bruker) for pre-concentration and desalting in 2% (v/v) acetonitrile with 0.1% (v/v) formic acid, then injected into the analytical column. The reverse phase nanoLC eluent was subjected to positive ion nanoflow electrospray analysis in an IDA mode. For data independent acquisition (SWATH), 10 μl of each sample was treated as for the IDA samples, with the reverse phase nanoLC eluent subjected to positive ion nanoflow electrospray in a data independent acquisition mode. For SWATH-MS, m/z window sizes were determined based on precursor m/z frequencies (m/z 400–1250) from the IDA data. In SWATH mode, a TOFMS survey scan was acquired (m/z 350–1,500, 0.05 sec) then 60 predefined m/z ranges were sequentially subjected to MS/MS analysis. MS/MS spectra were accumulated for 60 ms in the mass range 350–1,500 with optimised rolling collision energy.

#### Data processing and analysis

LC-MS/MS data from the IDA experiments were searched using ProteinPilot (v4.2; AB Sciex) against the ToxoDB GT1 proteome (ToxoDB.org). SWATH data were extracted using PeakView (v2.1) with the following parameters: the six most intense fragments of each peptide were extracted from the SWATH data sets, with shared and modified peptides excluded. Peptides with confidence ≥ 99% and FDR ≤ 1% were used for quantitation. SWATH protein peak areas were analysed using an in-house Australian Proteome Analysis Facility (APAF) program. Protein peaks were normalised to total peak area for each run, and were subjected to statistical analysis to compare relative protein peak areas between the sample groups. The data for each identified protein is presented in **S1 Table**.

### Dual luciferase reporter assays

To measure nanoLUC and fLUC activity in dual luciferase reporter strains, we cultured parasites in 25 cm^2^ tissue culture flasks in the required growth medium. Before parasite inoculation, host cells and parasites were both were washed twice with PBS to remove residual media. Parasites were cultured for between 38 and 42 hr, over which time all remained intracellular. Parasites grown in 10 μM Arg exhibited slower growth than at other [Arg] across this timeframe. To compensate for this, we inoculated more parasites into flasks containing 10 μM Arg. On the day of the experiment, parasites were liberated from host cells by passage through a 26 gauge needle. Host cell debris were removed by filtering through a 3 μm polycarbonate filter, and parasites were pelleted by centrifugation at 1,500 × *g* for 10 min. Parasites were resuspended to 1–2 × 10^7^ parasites/ml in PBS and 25 μl of parasite suspension was added to wells of an OptiPlate-96 opaque, white 96-well plate (PerkinElmer). To measure nanoLUC and fLUC luminescence, we used the NanoGlo Dual-luciferase reporter assays system (Promega, N1610). First, we measured fLUC activity by adding 25 μl ONE-Glo Ex Luciferase assay buffer with added substrate to wells containing parasites, incubating for 5 min, then reading on a FluoStar Optima plate reader (BMG Labtech) using the luminescence settings without an emission filter. Next, we measured nanoLUC activity by adding 25 μl NanoDLR Stop & Go assay buffer containing 1:100 diluted substrate to the parasite suspension, incubating for 5 min, then reading luminescence using the same settings as for fLUC. In each assay, we included a ‘no parasite’ control (25 μl PBS), which was subtracted from the luminescence readings of the parasite-containing wells before subsequent data analysis. To measure nanoLUC and fLUC activities in mouse organs, infected and uninfected mice were euthanised by cervical dislocation. Prior to organ harvest, intraperitoneal lavage was performed by injecting 5 ml ice-cold PBS into the intraperitoneal cavity using a 26 G needle, mixing peritoneal cavity content and subsequent aspiration of the content using 20 G needle. Next, incisions were made to open the chest cavity without damaging any organs. The spleen, liver and kidneys were harvested and placed in 2 ml ice-cold PBS. Next, the lungs were perfused by injection of 10 ml of ice-cold PBS into mouse heart ventricles. The heart, lung and brain were subsequently harvested and kept in 2 ml of ice-cold PBS. All samples were kept on ice until luminescence measurements. For luminescence measurements, all organs were homogenised using a dounce homogeniser. 25μl aliquots of each of the crude homogenate samples were transferred in duplicate into wells of an OptiPlate-96 opaque, white 96-well plate. NanoLUC and fLUC measurements were performed as described above. Luminescence measurements in the heart and brain of infected mice were found to be at background levels, and were not analysed further.

### Arg uptake experiments

Experiments to measure uptake of [^14^C]Arg through *Tg*ApiAT1 were performed as described previously [15,57]. Briefly, extracellular *T*. *gondii* parasites were incubated in PBS containing 10 mM D-glucose, 0.1 μCi/ml [^14^C]Arg, 50 μM unlabelled Arg, and 80 μM unlabelled Lys for a range of times. The unlabelled Lys was added to inhibit Arg uptake through *Tg*ApiAT6-1. The reaction was stopped by centrifuging the parasites through an oil mix consisting of 84% (v/v) PM125 silicone fluid and 16% (v/v) light mineral oil. The incorporated radiolabel was measured using a liquid scintillation counter (Perkin Elmer). Timecourse data were fitted by a single exponential function.

### Statistics and reproducibility

Unless described otherwise in the figure legends, all quantitative data are presented as mean ± SD of three or more independent experiments. All non-quantitative data (western blots, plaque assays) displayed are representative images of multiple independent experiments, with the number of experiments listed in the figure legends. Graphs were plotted using GraphPad Prism, and statistics were also undertaken in GraphPad Prism. Details of statistics are provided in the figure legends.

## Supporting information

S1 Fig Uncropped images of all anti-*Tg*ApiAT1-HA_3_ western blots included in the manuscript. Blots show the presence of the major *Tg*ApiAT1-HA_3_ protein species between the 40 and 50 kDa markers. Many images show additional minor protein species at higher molecular masses. The figure for which each image was utilized is indicated below each blot.
(TIF)

S2 Fig Luminescence readings of nanoLUC and fLUC expressing parasites grown at a range of [Arg]. NanoLUC and fLUC luminescence in a parasite strain expressing nanoLUC from the *Tg*ApiAT1 5’ region (*apiAT1* 5’-nanoLUC; red) and fLUC from the α-tubulin 5’ region (*tub* 5’-fLUC; blue), or a strain expressing nanoLUC from the α-tubulin 5’ region (*tub* 5’-nanoLUC; black), grown at a range of [Arg]. Luminescence is expressed as a percent of the luminescence at the 10 μM Arg condition for both nanoLUC and fLUC measurements. Data points represent the mean ± SD of nine independent experiments in the *apiAT1* 5’-nanoLUC/*tub* 5’-fLUC strain, and the mean ± SD of four independent experiments in the *tub* 5’-nanoLUC strain.
(TIF)

S3 Fig The 5’ region of *Tg*ApiAT1 mediates regulation in response to a range of nutrients. NanoLUC and fLUC luminescence readings in a parasite strain expressing nanoLUC from the *Tg*ApiAT1 5’ region (red) and fLUC from the α-tubulin (tub) 5’ region (blue), and grown at a range of (**A**) [Lys], (**B**) [Tyr], (**C**) [Gln], and (**D**) D-glucose. Luminescence is expressed as a percent of the luminescence at the highest tested concentration of each nutrient for both nanoLUC and fLUC measurements. Data points represent the mean ± SD of three independent experiments for each nutrient.
(TIF)

S4 Fig Disruption of *Tg*ApiAT1 impairs parasite growth in DME but not RPMI. 500 WT (RHΔ*hxgprt*/*apiAT1* 5’-nanoLUC/*tub*-fLUC; left) or *apiAT1*^*Δ54–534*^ (RHΔ*hxgprt*/*apiAT1* 5’-nanoLUC/*tub*-fLUC/*apiAT1*^*Δ54–534*^; right) parasites were inoculated into 25 cm^2^ tissue culture flasks containing either RPMI (top) or DME (bottom) and cultured for 8 days before staining with crystal violet to reveal plaque formation. Images are from a single experiment, and are representative of three independent experiments.
(TIF)

S5 Fig Schematic of the *Tg*ApiAT1 transcript. The *Tg*ApiAT1 gene encodes a transcript of 3,345 bp following splicing of the 203 bp intron. The main open reading frame (ORF) is encoded by 1,602 bp. The 5’ untranslated region (UTR) is 703 bp, and encodes an 99 bp upstream ORF (uORF) that participates in Arg-dependent regulation of the main ORF. The positions of the 5’ cap, the AUG start codons of the uORF and main ORF peptides, the UAA and UGA stop codons of the uORF and main ORF, the 3’ UTR, and the poly-adenylate (poly(A)) tail of the transcript are also shown.
(TIF)

S1 Table Data from the SWATH-MS proteomic analysis. **Tab 1.** Averaged data from all replicates, indicating the ToxoDB ID, the number of peptides used in the analysis of each protein, *P* value, -log_10_ *P* value, the average fold change in the high vs low [Arg] conditions, the average fold change in the low vs high [Arg] conditions, the log_2_ fold change in the low vs high [Arg] condition, and the protein annotation. **Tab 2.** The data from each replicate of the experiment. H = 1.15 mM Arg; L = 50 μM Arg.
(XLSX)

S2 Table Sequences of the primers and gBlocks used in this study. (DOCX)
