# Supplementary figures and images for: Substrate-mediated regulation of the arginine transporter of Toxoplasma gondii
Source: PLoS Pathog. 2021 Aug 5;17(8):e1009816. doi: 10.1371/journal.ppat.1009816 (PMC8370653; doi:10.1371/journal.ppat.1009816)

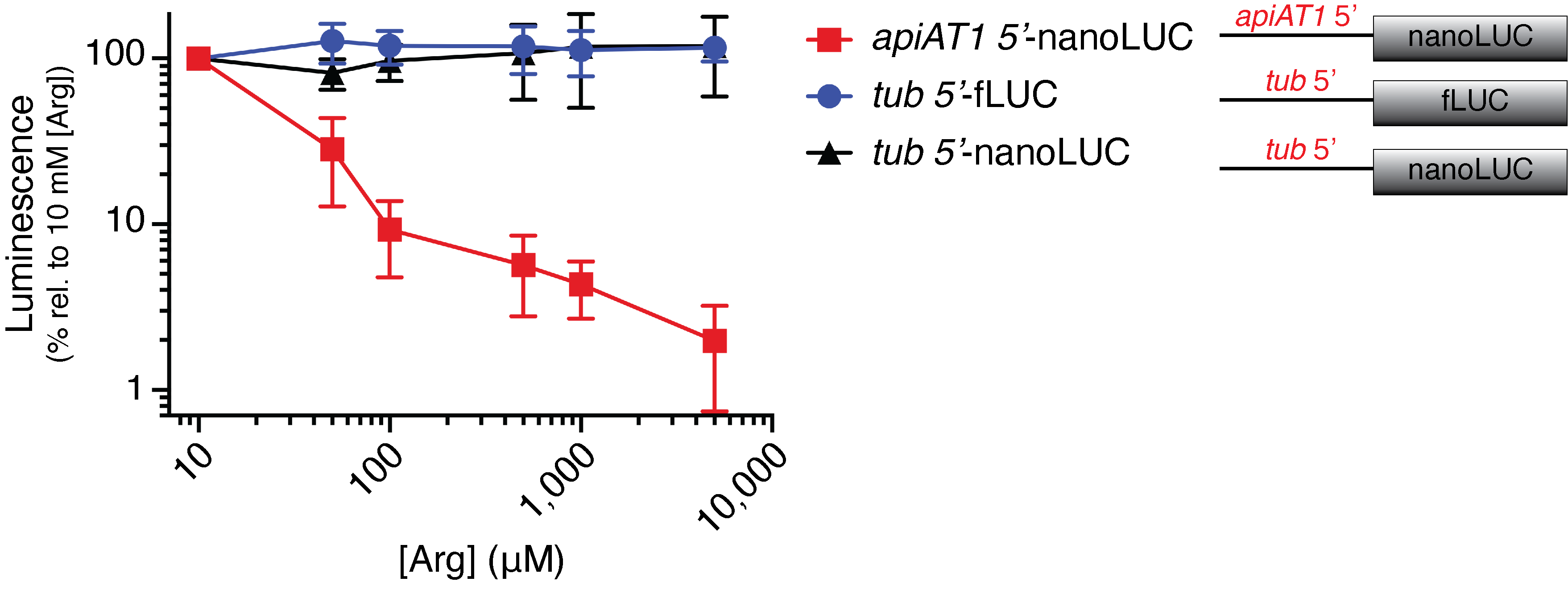

Supplement: S2 Fig — NanoLUC and fLUC luminescence in a parasite strain expressing nanoLUC from the TgApiAT1 5’ region (apiAT1 5’-nanoLUC; red) and fLUC from the α-tubulin 5’ region (tub 5’-fLUC; blue), or a strain expressing nanoLUC from the α-tubulin 5’ region (tub 5’-nanoLUC; black), grown at a range of [Arg]. Luminescence is expressed as a percent of the luminescence at the 10 μM Arg condition for both nanoLUC and fLUC measurements. Data points represent the mean ± SD of nine independent experiments in the apiAT1 5’-nanoLUC/tub 5’-fLUC strain, and the mean ± SD of four independent experiments in the tub 5’-nanoLUC strain. (TIF) [file ppat.1009816.s002.tif]

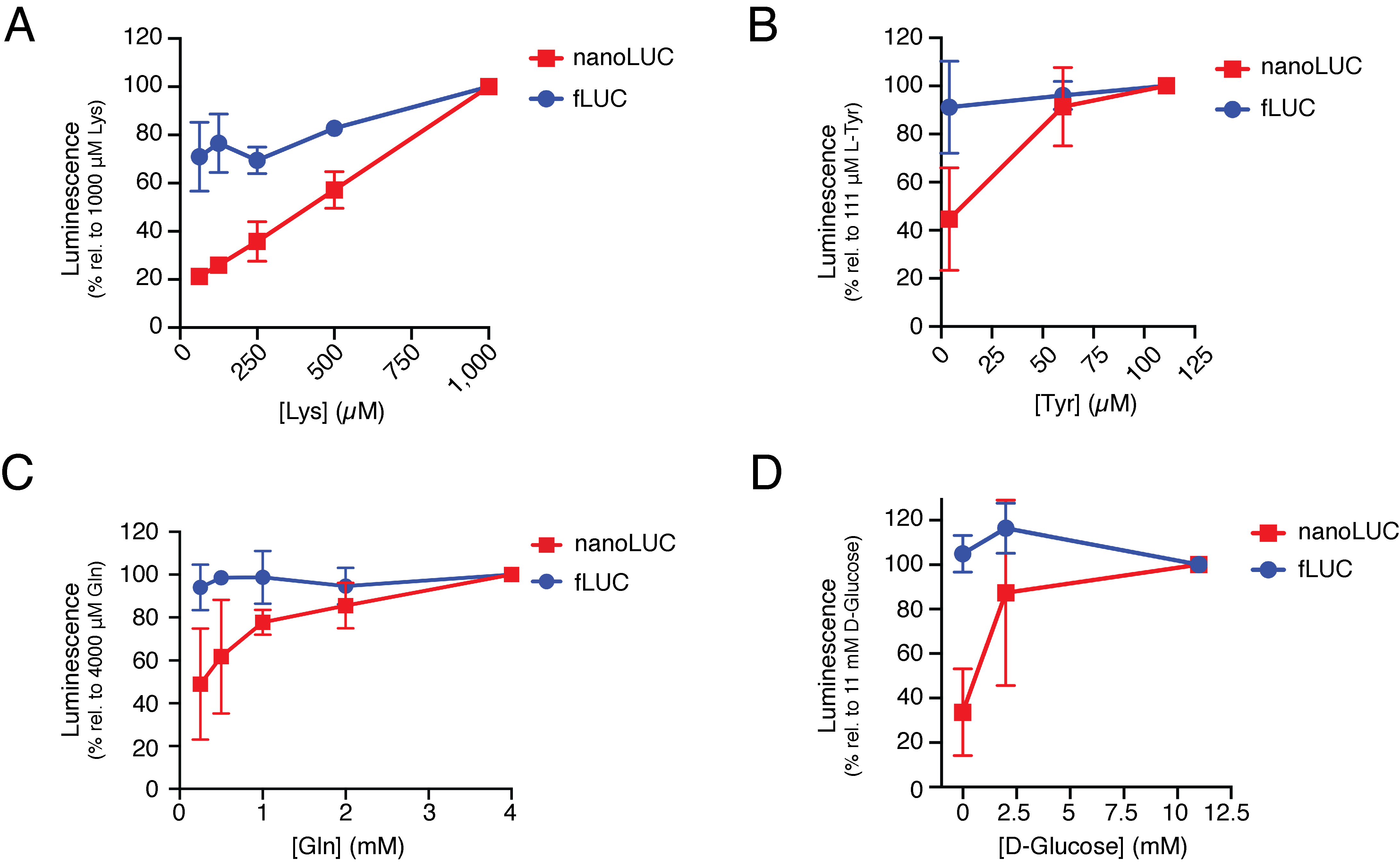

Supplement: S3 Fig — NanoLUC and fLUC luminescence readings in a parasite strain expressing nanoLUC from the TgApiAT1 5’ region (red) and fLUC from the α-tubulin (tub) 5’ region (blue), and grown at a range of (A) [Lys], (B) [Tyr], (C) [Gln], and (D) D-glucose. Luminescence is expressed as a percent of the luminescence at the highest tested concentration of each nutrient for both nanoLUC and fLUC measurements. Data points represent the mean ± SD of three independent experiments for each nutrient. (TIF) [file ppat.1009816.s003.tif]

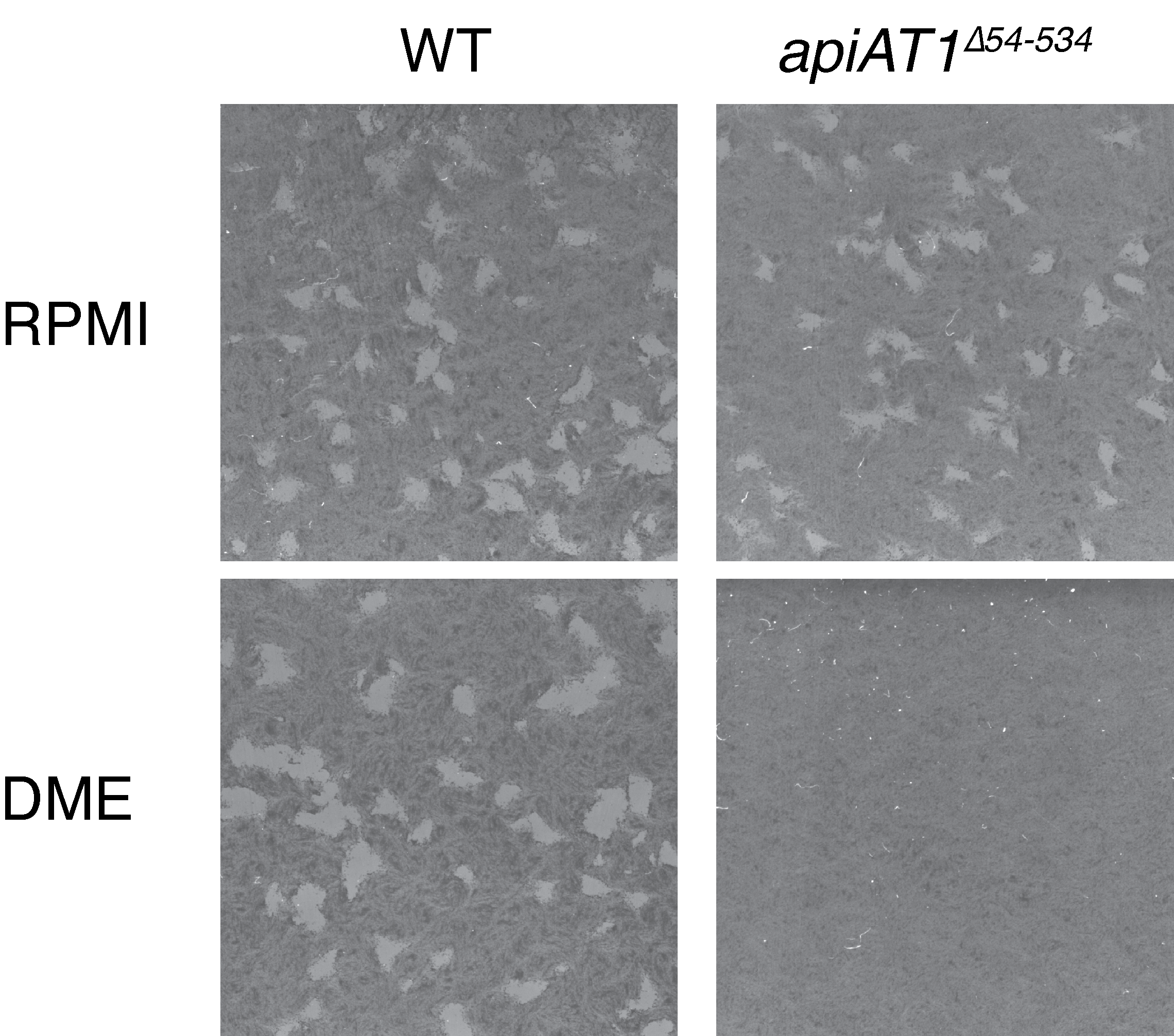

Supplement: S4 Fig — 500 WT (RHΔhxgprt/apiAT1 5’-nanoLUC/tub-fLUC; left) or apiAT1Δ54–534 (RHΔhxgprt/apiAT1 5’-nanoLUC/tub-fLUC/apiAT1Δ54–534; right) parasites were inoculated into 25 cm2 tissue culture flasks containing either RPMI (top) or DME (bottom) and cultured for 8 days before staining with crystal violet to reveal plaque formation. Images are from a single experiment, and are representative of three independent experiments. (TIF) [file ppat.1009816.s004.tif]

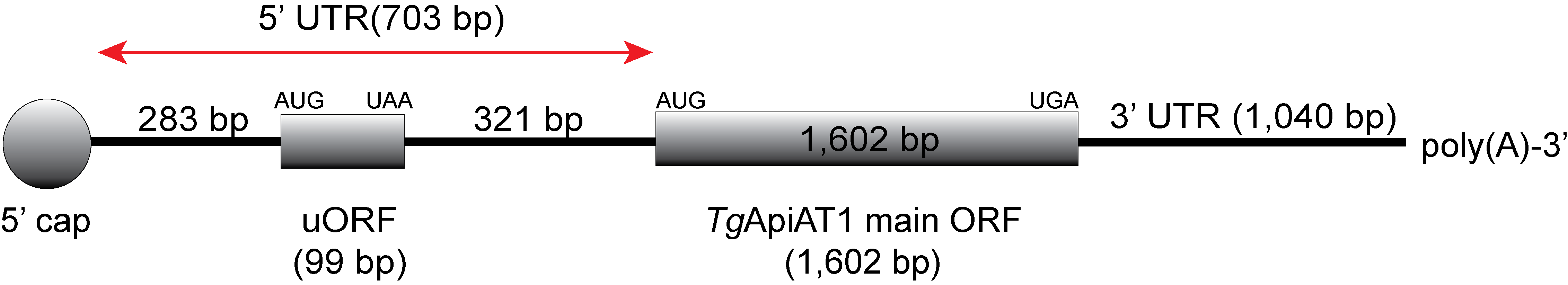

Supplement: S5 Fig — The TgApiAT1 gene encodes a transcript of 3,345 bp following splicing of the 203 bp intron. The main open reading frame (ORF) is encoded by 1,602 bp. The 5’ untranslated region (UTR) is 703 bp, and encodes an 99 bp upstream ORF (uORF) that participates in Arg-dependent regulation of the main ORF. The positions of the 5’ cap, the AUG start codons of the uORF and main ORF peptides, the UAA and UGA stop codons of the uORF and main ORF, the 3’ UTR, and the poly-adenylate (poly(A)) tail of the transcript are also shown. (TIF) [file ppat.1009816.s005.tif]
