# Supplementary material for: Substrate-mediated regulation of the arginine transporter of Toxoplasma gondii
Source: PLoS Pathog. 2021 Aug 5;17(8):e1009816. doi: 10.1371/journal.ppat.1009816 (PMC8370653; doi:10.1371/journal.ppat.1009816)
Supplement: S2 Table — (DOCX) [file ppat.1009816.s007.docx]

**S2 Table**. Sequences of the primers and gBlocks used in this study.

| Oligonucleotide name | Oligonucleotide sequence (5’ to 3’) |
| --- | --- |
| ApiAT1 3’ gRNA fwd | GACTTATTTTCATGCTGCATGTTTTAGAGCTAGAAATAGCAAG |
| Generic rvs | AACTTGACATCCCCATTTAC |
| ApiAT1 3’ edit fwd | GAAGACATTCGCAGTCGCGT |
| ApiAT1 3’ edit rvs | GCCGATTGAAGAGCCACAAC |
| fLUC fwd | GACTAGATCTGCGATCGCAAAATGGAAGACGCCAAAAACATAAAG |
| fLUC rvs | GATCCCTAGGCACGGCGATCTTTCCGCCCTTC |
| ApiAT1 5’ fwd | GACTACTAGTGAGCAAACAGTCACTTTAATGTGG |
| ApiAT1 5’ rvs | CATGGCGATCGCTATCCTGCAGGAACCTCCCGCGGGAACAGCA |
| ApiAT1 5’ UTR fwd | GATCCCTGCAGGAGTTCATTCTTTGAAAATATGCTCCAG |
| ApiAT1 5’ UTR rvs | CATGGCGATCGCAATGCCAACACGAATGAGATTCAAC |
| nanoLUC fwd | CATGGCGATCGCAAAATGGTCTTCACACTCGAAGATTTCGTTG |
| nanoLUC rvs | GATCCCTAGGTCCGCTACCACCTGAGCCTCCA |
| ApiAT1 uORF gRNA fwd | GACGACCATTTTTTCGGACGGTTTTAGAGCTAGAAATAGCAAG |
| ApiAT1 ∆uORF fwd | TCCAGTCTTTTCAGTAAAGGAGAACCAATCTGTGTGCGGGCGCGTCCGAAAAATTGGTCGTCGAATTGATTTCGCAGTACCCTTTCTTGA |
| ApiAT1 ∆uORF rvs | TCAAGAAAGGGTACTGCGAAATCAATTCGACGACCAATTTTTCGGACGCGCCCGCACACAGATTGGTTCTCCTTTACTGAAAAGACTGGA |
| uORF D19N fwd | CACTAGGGATaACTACCTCGATATTG |
| uORF D19N rvs | CAACCAGTCAAGAAAGGG |
| Tub 5’ fwd | GACTACTAGTGCATACATTATACGAAGTTATTGCTAGAATG |
| Tub 5’ rvs | CATGGCGATCGCAAAAGGGAATTCAAGAAAAAATGC |
| ApiAT1 qrt int fwd | CTCTCGACGATTCCTTGTCTGCT |
| ApiAT1 qrt int fwd | GAAATACTGGGCCACCACGCT |
| ApiAT1 qrt 3’ UTR fwd | CATGCGTTGTGGCTCTTCAATC |
| ApiAT1 qrt 3’ UTR rvs | CCAACTGTTTCTGCATCGTCGT |
| Tub qrt fwd | CGACGCCTTCAACACCTTCTTT |
| Tub qrt rvs | AGTTGTTCGCAGCATCCTCTTTC |
| GAPDH qrt fwd | TGGTGTTCCGTGCTGCGAT |
| GAPDH qrt rvs | AGCTTGCCGTCCTTGTGGC |
| *Tg*ApiAT1-HA_3_ gBlock | GAAGACATTCGCAGTCGCGTGCTGGAACTCAAAGCAGCACACGCTGCAGATGCAGCAGGAGGTGGTAGCGGTGGAGGTAGTTACCCGTACGACGTCCCGGACTACGCTGGCTATCCCTATGATGTGCCCGATTATGCGTATCCTTACGATGTTCCAGATTATGCCTGAAAATAAGTCCCGCACCTGGCGCATGCGTTGTGGCTCTTCAATCGGC |
| *Tg*ApiAT1/∆uORF 5’UTR gBlock | AGTTCATTCTTTGAAAATATGCTCCAGCGTCATCGTTTACTGCTTTCAGAATTGCAAAGCACTTTCGAACGATTTTACAAGGTGTAAAGACGGGTATTCTCAAGGTGGCGCAGCCAGAGTTCCTAGCAGCTTGCGAACGCACCACCACGTGGAATTGCTTCCGGGAGAGCTATCCTGTTGCCTGCTTCCGCTTTGTGGCCATCTTTAGATTTTTCATTTCTTCTCAGCGCTCCAGTCTTTTCAGTAAAGGAGAACCAATCTGTGTGCGGCCGCGTCCGAAAAATTGGTCGTCGAATTGATTTCGCAGTACCCTTTCTTGACTGGTTGCACTAGGGATGACTACCTCGATATTGCGGTCTGGAAGTCCGTAGCTCTCATATGCTAACTTTCCTCAAAAAGACATATTTTTTGTTTGTGCTGTGTTGGCACTATTGTGTTTCTTAATTATTTAGGTGTTTGTTTTTTCGTTACCCATCAGTGGACGCGCCGGCTTTGCTCGCTGGCGTGGCCGTCTCCCAGCTTCTGCGTTGTCCAATAACACCGGTGCTGTCTATTTCTGCGCTCATTTCGCAAGAATCGCGGAGAGTTTCATCTCTTTTGCCCGTATCTTGTCGTTTTCTTAAGAATCGAAGAGGCTATCTTCGCTGCGACTTTAGCCTTTCTCGGTCCGCCCTTGCTGTTGAATCTCATTCGTGTTGGCATT |
| *Tg*ApiAT1/ScAAP uORF 5’UTR gBlock | AGTTCATTCTTTGAAAATATGCTCCAGCGTCATCGTTTACTGCTTTCAGAATTGCAAAGCACTTTCGAACGATTTTACAAGGTGTAAAGACGGGTATTCTCAAGGTGGCGCAGCCAGAGTTCCTAGCAGCTTGCGAACGCACCACCACGTGGAATTGCTTCCGGGAGAGCTATCCTGTTGCCTGCTTCCGCTTTGTGGCCATCTTTAGATTTTTCATTTCTTCTCAGCGCTCCAGTCTTTTCAGTAAAGGAGAACCAATCTGTGTGCGGCCGCGTCCGAAAAAATGTTTAGCTTATCGAACTCTCAATACACCTGCCAAGACTACATATCTGACCACATCTGGAAAACTAGCTCCCACTAACTTTCCTCAAAAAGACATATTTTTTGTTTGTGCTGTGTTGGCACTATTGTGTTTCTTAATTATTTAGGTGTTTGTTTTTTCGTTACCCATCAGTGGACGCGCCGGCTTTGCTCGCTGGCGTGGCCGTCTCCCAGCTTCTGCGTTGTCCAATAACACCGGTGCTGTCTATTTCTGCGCTCATTTCGCAAGAATCGCGGAGAGTTTCATCTCTTTTGCCCGTATCTTGTCGTTTTCTTAAGAATCGAAGAGGCTATCTTCGCTGCGACTTTAGCCTTTCTCGGTCCGCCCTTGCTGTTGAATCTCATTCGTGTTGGCATT |
| *Tg*ApiAT1/hybrid uORF 5’UTR gBlock | AGTTCATTCTTTGAAAATATGCTCCAGCGTCATCGTTTACTGCTTTCAGAATTGCAAAGCACTTTCGAACGATTTTACAAGGTGTAAAGACGGGTATTCTCAAGGTGGCGCAGCCAGAGTTCCTAGCAGCTTGCGAACGCACCACCACGTGGAATTGCTTCCGGGAGAGCTATCCTGTTGCCTGCTTCCGCTTTGTGGCCATCTTTAGATTTTTCATTTCTTCTCAGCGCTCCAGTCTTTTCAGTAAAGGAGAACCAATCTGTGTGCGGCCGCGTCCGAAAAAATGGTCGTCGAATTGATTTCGTTTAGCTTATCGAACTCTCAATACACCTGCCAAGACTACATATCTGACCACATCTGGAAAACTAGCTCCCACATATGCTAACTTTCCTCAAAAAGACATATTTTTTGTTTGTGCTGTGTTGGCACTATTGTGTTTCTTAATTATTTAGGTGTTTGTTTTTTCGTTACCCATCAGTGGACGCGCCGGCTTTGCTCGCTGGCGTGGCCGTCTCCCAGCTTCTGCGTTGTCCAATAACACCGGTGCTGTCTATTTCTGCGCTCATTTCGCAAGAATCGCGGAGAGTTTCATCTCTTTTGCCCGTATCTTGTCGTTTTCTTAAGAATCGAAGAGGCTATCTTCGCTGCGACTTTAGCCTTTCTCGGTCCGCCCTTGCTGTTGAATCTCATTCGTGTTGGCATT |
