# Supplementary material for: Substrate-mediated regulation of the arginine transporter of Toxoplasma gondii
Source: PLoS Pathog. 2021 Aug 5;17(8):e1009816. doi: 10.1371/journal.ppat.1009816 (PMC8370653; doi:10.1371/journal.ppat.1009816)
Supplement: Attachment — Submitted filename: Response to reviewer comments.pdf [file ppat.1009816.s008.pdf]

Response to PLOS Biology reviewer comments

Below is a point-by-point response to reviewer comments on the original manuscript that was reviewed in PLOS Biology. We have numbered the reviewer questions to allow us to cross-reference responses to similar queries from the reviewers. Reviewer comments are in black and our responses in blue.

Reviewer 1.

The MS by Rajendran et al. reports the negative regulation of an Arginine transporter by its substrate, Arginine. The authors characterize this regulation and find a sequence situated upstream of the gene encoding for the regulated transporter as the responsible for this regulation. The phenomenon is interesting, however I am not sure if it has the degree of novelty required for PLoS-Biology. As in fact, this is a decision of the Editor, I will focus on the sceince. The rational in general is quite clear, however, I have several serious concerns that are comented below. Taking them into account, I do not recomend the publication of the MS in its present form.

Major points:

Results:

1. General: it is recommended nowadays as a good practice to show at least as supplementary material the whole western blotting results and not only the region of the membrane where the bands of interest have been revealed. I strongly recommend the authors to include the whole register of WBs. Please refer to my comment on Fig. 3 to understand my concern.

We have included a supplementary figure (S1 Fig) that includes the 'raw' images of all the *Tg*ApiAT1-HA$_3$ western blots depicted in the manuscript (see also our more detailed response to the comment 9 by Reviewer 1).

2. Fig. 1: Panel A: as the experiment is repeated in Panel B, this panel is irrelevant.

We respectfully disagree. Panel A was performed in a Type I strain of *T. gondii* (RH background) whereas Panel B was performed in a Type II strain (Prugniaud background). The majority of subsequent experiments were performed in Type I strain, and it is therefore important that we include Figure 1A. RH strain parasites do not readily undergo differentiation to bradyzoite stages, whereas Prugniaud strain parasites do. Since arginine depletion has been linked to bradyzoite differentiation, it was important to establish whether the regulation of *Tg*ApiAT1 is tied to a general bradyzoite differentiation response. The data from Figure 1B show that it isn't, and it is therefore critical for us to include these data. An additional conclusion from these experiments is that the Arg-dependent regulation of *Tg*ApiAT1 is observed in both Type I and Type II strain parasites. We have added this observation to the text, and further expanded the description of this experiment in response to a comment from feedback to the BioRxiv posting of our manuscript (see later). Specifically, we now state:

"Low [Arg] conditions have been linked to formation of the latent bradyzoite stage of *T. gondii* [ref]. We HA-tagged *Tg*ApiAT1 in Type II Prugniaud strain *T. gondii* parasites, which readily form bradyzoites, and cultured parasites for two days at a range of initial [Arg] in the culture medium. As for Type I parasites, expression of *Tg*ApiAT1-HA$_3$ increased with decreasing [Arg] (**Fig 1B**). To test whether *Tg*ApiAT1-HA$_3$ abundance changed upon bradyzoite differentiation, we induced bradyzoite differentiation by incubating parasites for six days in high pH conditions in a medium containing 1.15 mM Arg. We observed increased expression of the bradyzoite-specific antigen *Tg*BAG1 and a bradyzoite-specific green fluorescence protein (GFP), and decreased expression of the tachyzoite specific antigen *Tg*SAG1, verifying that bradyzoite differentiation had occurred (**Fig 1B**). Notably, we observed no induction of *Tg*ApiAT1-HA$_3$ expression in these conditions (**Fig 1B**). These data indicate that *Tg*ApiAT1 regulation is not related to the general bradyzoite differentiation response of the parasite, and that Arg-dependent *Tg*ApiAT1 regulation occurs in both Type I and Type II strains of *T. gondii*. The short timeframe over which we cultured parasites in depleted [Arg] means that our data do not rule out the possibility that Arg depletion causes bradyzoite differentiation, or rule out a role for *Tg*ApiAT1 in mediating bradyzoite differentiation." (Lines 97-112)

3. Additionally, in panel B detected TgApiAT1-HA3 with more sensitivity (it was detectable with 100 micromolar Arg) and included a control without Arg (which in fact shoed a higher expression of the analysed protein than any other in the presence of Arg, which is weird). Does it all make sense?

In both Figures 1A and 1B we observe lower levels of *Tg*ApiAT1 expression when parasites are cultured at 50 μM than at 10 μM. As the reviewer notes, at 100 μM, we see barely detectable levels of *Tg*ApiAT1-HA3 when Type II parasite are cultured in 100 μM Arg, whereas we don't detect any clear expression in Type I parasites cultured in 100 μM Arg. Although these (very slight) differences could be due to strain-to-strain variation, it is more likely to occur for technical reasons (e.g. differences in exposure times of the different blots to film) or perhaps from variations between experimental replicates (e.g. small differences in the time parasites have been cultured before harvesting). Regardless, the take-home message from these experiments is that *Tg*ApiAT1 abundance is regulated by [Arg].

4. Is there another Arg transporter with a highest Km?

As we describe in Figure 4, *T. gondii* has a cationic amino acid transporter called *Tg*ApiAT6-1 that is able to transport Arg. In response to Comment 13 from Reviewer 3, we have uploaded a manuscript (Fairweather et al, 2021) that describes our studies on *Tg*ApiAT6-1 to BioRxiv (https://www.biorxiv.org/content/10.1101/2021.06.25.450001v1) and reference this study at various points in our manuscript. To answer the reviewer's question directly, the Fairweather et al manuscript reveals that *Tg*ApiAT6-1 has a K_M for Arg of ~750 μM, which is indeed greater than the K_M for *Tg*ApiAT1 (88 μM; Rajendran et al, PMID 28205520). As we describe in the Fairweather et al manuscript, *Tg*ApiAT6-1 is able to transport both Lys and Arg, and has a higher affinity (i.e. lower K_M) for Lys than Arg. We have modified the text to state this explicitly: "*T. gondii* parasites have two Arg transporters: the selective Arg transporter *Tg*ApiAT1 and the general cationic amino acid transporter *Tg*ApiAT6-1, which has a higher affinity for L-lysine (Lys) than Arg". (Lines 136-138)

5. Panel C and D: they show a relatively quick Arg-dependent regulation. I think they could be joined in the same panel. I would suggest to include a graphic with band densitometries to have a relative quantification of the protein for this.
We have chosen not to join the data in a single panel, since these are quite separate experiments. We appreciate the suggestion on undertaking densitometric analyses, and note that Reviewer 3 also requested that we quantify the $t_{0.5}$ for up- and down-regulation of *Tg*ApiAT1-HA$_3$ following the media switch. A challenge in performing densitometry on the original images is that *Tg*ApiAT1-HA$_3$ was not detectable at numerous time points, and therefore could not be quantified. We have therefore repeated these experiments and developed our western blots using a BioRad ChemiDoc imaging system, which takes digital chemiluminescence images. The digital images provide greater sensitivity and resolution than the x-ray film images we used in the initial manuscript. This enables more accurate quantification of band intensities in the western blots. Additionally, we repeated the "high-to-low" switch using medium containing 0 μM Arg, since this facilitates a maximal parasite response to Arg depletion, and therefore more accurately represents the time frame of changes to *Tg*ApiAT1 abundance (note that we did not use 0 μM Arg in the 'low-to-high' switch, since parasite proliferation across the two days of incubation in these experiments is inhibited in medium lacking Arg). For both switches, we included earlier timepoints to obtain a more resolved picture of the regulation response.

Based on quantification of the 'high-to-low' switch experiment now shown in Figure 1C, we observe an rapid increase in *Tg*ApiAT1-HA$_3$ abundance following the medium switch, with *Tg*ApiAT1-HA3 abundance doubling after 30 min and reaching a significant, ~8-fold increase after 4 hr. We observe a decrease in *Tg*ApiAT1-HA$_3$ abundance in the 'low-to-high' medium switch from 8 hrs post-switch (where abundance is 24% of that observed in the parasites cultured in the low Arg medium throughout the experiment; Figure 1D), continuing to ~7% of the initial amount after 24 hr. Together, these new data support our initial assertions that *T. gondii* parasites change the abundance of *Tg*ApiAT1 in response to the [Arg] they encounter in their growth medium, with parasites able to upregulate *Tg*ApiAT1-HA$_3$ expression in response to Arg limitation more rapidly than they are able to downregulate *Tg*ApiAT1-HA$_3$ when [Arg] becomes abundant. We have modified the description of these experiments in the text accordingly. (Lines 113-127)

6. Figure 1. I would like to state that just a time-course experiment does not constitute a kinetic analysis, so I would mention these experiments as time-course (I suggest to change this, in line 84 for example).
Changed as suggested. Now reads: "To assess the timeframe of the Arg-dependent regulation of *Tg*ApiAT1-HA$_3$…" (changes underlined; Line 113)

7. Line 100: the authors mention a measurement of initial rate of Arg uptake. Looking at Fig. S1 it is not possible to state that measurements at 2.5 min correspond to initial rates. This should be shown by making measurements at very short times (at least 15 s, at intervals of 15 – 30 s until reach the time of 2.5 min) and demonstrate that the obtained rates increase in an approximately linear-way during the whole lapse of time. If the authors do not show linearity until 2.5 min, they should not mention initial rates.
We agree with the reviewer's points on this. We have modified this figure to remove the initial rate measurements (*i.e.* the original Figure 1F), and include the timecourse uptake data (originally Figure S1, now Figure 1F). We also expand the description of this experiment in the results section so that readers are clear on how the experiment was performed. These data enable us to conclude that there is a decrease in *Tg*ApiAT1-dependent Arg uptake in parasites grown in medium containing higher [Arg], consistent with a decrease in the abundance of the *Tg*ApAT1 transporter (as shown in other panels of this figure).

8. Lines 130-131: it would be useful if the authors provides here a rationale for choosing Lys for example mentioning the explanation given later in lines 151-156 and citing reference 15).
As suggested, we moved part of the sentence from Lines 151-156 to this section: "Our previous study revealed a connection between the uptake of Arg and Lys in *T. gondii* [ref]." (Line 175). The later sentence has been modified to read: "In a previous study, we demonstrated the presence of a cationic amino acid transporter that has a higher affinity for Lys than for Arg [ref]" (Lines 197-199)

9. Figure 3: the antibody reveals a secondary band (a bit above 60 kDa) that apparently is regulated in a similar

way that TgApiAT1-HA3. Could the authors explain this? Does it correspond to a co-regulated protein recognized by the anti-HA3 antibody? If so more controls are required.
As suggested by the reviewer in their general comments, we now include the ‘uncropped’ *Tg*ApiAT1-HA_3 western blots of all the images we use in the manuscript figures as S1 Fig. As is apparent from that supplementary figure, we often but not always observe the minor higher molecular mass species that the reviewer mentions. As outlined in the methods, the western blots were performed with anti-HA antibodies, which in theory should detect only the HA-tagged *Tg*ApiAT1-HA3 protein. We suspect that the higher molecular mass species represent isoforms of *Tg*ApiAT1-HA3 that migrate more slowly by SDS-PAGE than the main isoform. This could, for example, represent post-translationally modified *Tg*ApiAT1 protein, or could be related to experimental variability in SDS binding to this hydrophobic protein (e.g. Rath et al, 2009; PMID 19181854). We have added the following text to the manuscript that describe the existence of these minor species, also pointing out that these exhibit similar regulation to the major protein species: “In many anti-*Tg*ApiAT1-HA_3 western blots, we observed additional minor higher molecular mass species which exhibited a similar Arg-dependent response (**S1 Fig**). These were not investigated further.” (Lines 93-96)

10. In vivo experiment: T. gondii parasites modulate TgApiAT1 expression in vivo. This experiment is interesting and could be informative, however it is difficult to interpret without information about the levels of Arg in the evaluated tissues. The authors claim that they are different but I could not find any information about how are these differences. It is almost impossible to make a conclusion about this with a more precise information about Arg concentrations in the analized tissues.
We attempted to determine arginine amounts in tissues harvest from both *T. gondii* infected and uninfected mice, but were unable to obtain consistent, reproducible data. There is, however, considerable evidence that Arg concentrations vary in different organs of mammals. Specifically, those studies have shown that Arg levels are highest in organs that serve as major sites of arginine synthesis (e.g. kidneys), and lower in organs where Arg catabolism is prominent (e.g. liver). These observations are in line with our study, which predicts that *T. gondii* parasites encounter higher levels of Arg in the kidneys than in the liver. We have modified the text to explicitly mention the limitation of not having determine [Arg] in organs themselves, and we have expanded the discussion on what is known about how [Arg] varies in different organs of mammals. We state:

“Using a novel, Arg-dependent dual luciferase-expressing parasite strain, we demonstrate that parasite expression of *Tg*ApiAT1 varies between different organs during a mouse infection (**Fig 5**). This is consistent with parasites encountering different Arg concentrations in the different organs they infect. We attempted to determine Arg levels in these various organs experimentally, but were unable to obtain reproducible data. Nevertheless, Arg metabolism is known to differ in different organs of mice and other mammals. For example, kidneys are a major site of Arg synthesis [ref], whereas liver cells have a high activity of the arginine degrading enzyme arginase [ref]. This is reflected in the [Arg] in these organs, with Arg levels in kidneys being higher than in the liver in many mammals, including mice and other rodents [refs]. In concurrence with these studies, we demonstrate that *Tg*ApiAT1 expression is significantly higher in the liver than in the kidneys (**Fig 5**). In addition to a likely role for Arg in modulating *Tg*ApiAT1 expression *in vivo*, our *in vitro* data indicate that Lys and other nutrients also contribute to *Tg*ApiAT1 regulation, and it is likely that the abundances of these nutrients contribute to *Tg*ApiAT1 regulation *in vivo*. Together, our data provide experimental evidence that parasites are encountering different nutrient conditions in different physiological niches in their hosts, and that they are able to sense and respond to these differences.” (Lines 351-366)

11. Fig. 5, legend: it is not clear to me how can be measured NanoLUC:fLUC ratios in WT parasites: I understand that they are a recombinant lineage expressing those reporters, so they are not WT.
We mean nanoLUC/fLUC-expressing parasites where nanoLUC is expressed from the 5’ UTR of *Tg*ApiAT1 found in wild type parasites (as opposed to the various modified 5’ UTRs we report later in the study). We have removed mention of “WT” this sentence to avoid any confusion, and now refer to these as “*apiAT1 5’*-nanoLUC:*tub 5’*-fLUC parasites” in the figure legend. (Lines 1007, 1012 and 1014)

Discussion:
12. The authors repeat most of results, and discuss poorly their findings.
Without specifics, we are unsure of exactly what the reviewer means. We have expanded the discussion to include numerous additional points to address point 10 by this reviewer and point 16 raised by Reviewer 3. We reiterate key results (typically at the beginning of paragraphs) as a starting point for the subsequent discussion.

13. They claim that the parasites sense Arg in their environment (which they demonstrate in the MS) and respond by regulating the abundance of the Arg transporter TgApiAT1. However, the negative regulation of the transporter by its substrate is unexpected unless another transporter is compensatory expressed (which seems to be the case from the cited Ref. 25). However, nothing is mention or even speculated about the affinities of these transporters for Arg (which could be the reason for a switch).

As we note in a previous response, and as we describe in Figure 4 and the related discussion, *T. gondii* contains a general cationic amino acid transporter (*Tg*ApiAT6-1) that has a lower affinity for Arg than *Tg*ApiAT1. We now refer to a manuscript in which we characterise the affinities of *Tg*ApiAT6-1 for Arg and Lys (Fairweather et al, *BioRxiv*, 2021). We demonstrate in Figure 4 that *Tg*ApiAT6-1 expression does not change in response to changes in [Arg] (or [Lys]). This leads to our hypothesis that *Tg*ApiAT6-1 is constitutively expressed, but will only take up sufficient Arg for parasite growth when [Arg] in the medium is higher relative to [Lys]. So if a parasite encounters an environment with low [Arg], they upregulate *Tg*ApiAT1 to compensate. We have extended our description of this hypothesis in the discussion as follows:
"We hypothesise that *Tg*ApiAT1 regulation enables *T. gondii* parasites to respond to different [Arg] that parasites encounter in different *in vivo* environments. When the [Arg] that parasites encounter in their *in vivo* environment is low, reduced Arg uptake through *Tg*ApiAT6-1 leads to decreased [Arg] in the parasite, and promotes an upregulation of *Tg*ApiAT1 expression. *Tg*ApiAT1 regulation enables parasites to modulate Arg uptake from the host cell, thereby exerting tight control over their intracellular [Arg]." (Lines 344-350)

14. Lines 319-321: "Our data indicate that parasites vary the expression of TgApiAT1 in different organs during a mouse infection (Figure 5), which may reflect differences in host Arg metabolism and, consequently, [Arg] in these organs" – there are a quantity of papers reporting metabolomics data in different mammalian tissues… the authors should (at least) mention some values for the evaluated tissues. Even more, they should measure Arginine in those tissues in infected and non-infected mice. Home-made reliable assays are easy to set up, and there are several commercial kits that could be used as well.

See our response to point 10 from this reviewer – we now note the limitation of not having established an assay to measure Arg levels in organs of infected and uninfected mice, and we have expanded the discussion around the evidence that Arg levels differ in different mammalian organs.

Minor points:
Abstract:
15. Lines 22-23: a luciferase-based 'biosensor' strain of T. gondii – I can imagine that authors refer to a recombinant lineage, not exactly a strain. Please clarify.
The term "strain" to describe genetically modified *T. gondii* parasites is widespread, and we have opted not to change how we refer to these strains/lines/lineages. We believe that we are clear in describing the genetic modifications we have undertaken to generate each of the strains/lines/lineages that we develop and characterise, both here (i.e. stating that we have generated a luciferase-expressing strain) and elsewhere in the manuscript.

Introduction:
16. Lines 40-42: "Although early studies suggested that parasite metabolism is 'hard-wired' and resistant to adapting to changes in nutrient conditions…" I suppose that authors meant "Although early studies suggested that parasite metabolism is 'hard-wired' and resistant to changes in nutrient conditions…" Please check.
Modified as the reviewer suggests.

17. Lines 64-65: Again, please check the use of the word "strain". I suggest lineage, since this consists of a genetic modification of a pre-existing strain.
See response to comments 15.

Results:
18. Lines 106, 113 and others: again, consider changing strain for lineage
As above

Reviewer #2:

Rajendran et al

This manuscript describes the regulation of a Arginine transporter in Toxoplasma and its function in growth. The authors report that the expression of the transporter is post-transcriptionally controlled by Arginine availability and appears to be due to an upstream ORF located in the transporter's 5 UTR. Finally, they report that expression of the transporter is dynamic within tissues and propose that this is due to variations in Arginine levels between tissues. Overall, the work is very well executed and for the most part properly interpreted. Besides some issues listed below, I am not sure the work's novelty and/or impact reaches a level one would expect for a publication in PLoS Biology. That, however, is an editorial decision.

Major Comments:

1. The primary limitation of this paper are the in vivo experiments. The authors report that TgAPIAT1 expression is dynamic within tissues and this is due to arginine availability, although it is not shown that arginine levels vary between tissues. However, the authors show that other nutrients can impact TgAPIAT1 expression and those may vary as well in the tissues. Thus, it isn't clear what is controlling transporter expression in the tissues. Another problem is that it's unclear how dynamic the parasite's transcriptome/proteome is between tissues and therefore not known how specific differences in TgAPIAT1 expression. These are, obviously, difficult questions to address and doing so would be beyond the scope of this paper. I would therefore suggest these experiments be removed from the paper.
We agree that our paper leaves many unanswered questions regarding the [Arg] that parasites encounter *in vivo*, and whether this is the sole contributor to regulating *Tg*ApiAT1 (as the reviewer notes, our *in vitro* data suggest that multiple nutrients contribute). As we note in our response to comment 10 from Reviewer 1, there is abundant evidence that [Arg] varies between different organs in mice and other mammals. These studies found that Arg levels were highest in organs like the kidneys (where our *in vivo* data indicate that *Tg*ApiAT1 is least abundant) and lowest in organs like the liver (where our *in vivo* data indicate *Tg*ApiAT1 is most abundant). The correlation between our findings and what is known in the literature suggests that *Tg*ApiAT1 levels are modulated primarily by [Arg] *in vivo*, with other nutrients having a lesser role. We also note that the extent of Arg-dependent *Tg*ApiAT1 regulation that we observe *in vitro* (>50-fold between high and low Arg conditions; Fig 2B) is considerably greater than the regulation we observe with other nutrients (~2 or 3-fold for Lys, Tyr, Gln and Glucose; Fig 3B, E, G and I). Additionally, we note that Arg-dependent regulation of *Tg*ApiAT1 occurs in the *opposite* manner to regulation by the other nutrients, with *Tg*ApiAT1 *increasing* in abundance when Arg becomes limiting. Final, we present evidence that the Lys-dependent regulation of *Tg*ApiAT1 occurs primarily because of decreased Arg uptake into the parasite via *Tg*ApiAT6-1 as [Lys] increases (Fig 4F). However, we do agree with the reviewer that the response is probably multi-factorial, and we have added some sentences to the discussion to reflect this (see below).

Regarding whether to remove the data from the manuscript, we believe that our study provides some valuable (and novel) tools for analysing the parasite response to changes in [Arg] (and potentially other nutrients) in different organs and across the course of an infection. Additionally, the *in vivo* experiments are validation that the *in vitro* responses of TgApiAT1 to [Arg] are meaningful in an infection model (see Fig 5B). We have therefore opted to leave these experiments as part of the manuscript. To address the reviewer's point, we have included additional lines in the discussion:

a) To point out that the *in vivo* response is probably multifactorial: "In addition to a likely role for Arg in modulating *Tg*ApiAT1 expression *in vivo*, our *in vitro* data indicate that Lys and other nutrients also contribute to *Tg*ApiAT1 regulation, and it is likely that the abundances of these nutrients contribute to *Tg*ApiAT1 regulation *in vivo*." (Lines 361-364)
b) To point out the value of the *in vivo* data, and place this in broader context: "Together, our data provide experimental evidence that parasites are encountering different nutrient conditions in different physiological niches in their hosts, and that they are able to sense and respond to these differences" (Lines 364-366)
c) To point out the differences in the direction of Arg-dependent regulation vs regulation in response to other nutrients: "Our study indicates that *Tg*ApiAT1 is regulated by the availability of a range nutrients, including glucose, and the amino acids Gln and Tyr, although the direction of regulation is inverse to the regulation observed in response to changes in [Arg] (with *Tg*ApiAT1 abundance decreasing upon limitation of these nutrients)" (Lines 377-380) … "It remains to be determined whether the mechanism mediating this general starvation response is similar to the specific Arg-dependent response." (Lines 389-390)

2. The identification of a putative uORF is extremely interesting and the data are compelling. But data are lacking showing that the uORF is in fact expressed. From what I can tell, ESTs maps to the genes apparent 5' UTR but I had a hard time navigating the RNAseq data. Regardless, experimental verification of the uORF is critical since this is a major finding of the paper. The optimal experiment to address this would be mass spec to identify the expressed peptide, which is understandably difficult. At the very least, RT-PCR or 5' RACE to demonstrate that the uORF is in fact expressed is needed.
The length of the *Tg*ApiAT1 5' UTR is 703 bp. This is based on RNAseq data (*i.e.* actual sequencing of the transcripts) available in www.toxodb.org (*e.g.* see, for example, the ToxoDB page for *Tg*ApiAT1 and links to the RNAseq sequence data within: https://toxodb.org/toxo/app/record/gene/TGME49_215490#Sequences). Because sequence data is available, we don't see the value in undertaking RT-PCR or 5' RACE.

We have attempted experiments to detect the uORF-encoded peptide by introducing an epitope tag into uORF (not included in the manuscript). We found that this abolished *Tg*ApiAT1-dependent regulation, which is perhaps not surprising given that our data indicate the sequence and possibly the length of the peptide encoded by the uORF is critical for mediating regulation (Fig 7). Fig 6B-C and the entirety of Fig 7 present evidence that support a role for the uORF for *Tg*ApiAT1 regulation. uORFs can modulate expression of downstream genes in a variety

of ways. For example, they can cause ribosome dissociation from the transcript, ribosome stalling on the transcript, or facilitate mRNA degradation. Our data are not conclusive on the mechanism of uORF-mediated regulation of *Tg*ApiAT1, but we present strong evidence that the peptide sequence of the *Tg*ApiAT1 uORF is important (Fig 7B, wherein we show that a single amino acid change, resulting from a single nucleotide change, almost completely ablates Arg-dependent *Tg*ApiAT1 regulation). This makes the *Tg*ApiAT1 uORF one of the few examples in nature where the encoded peptide is predicted to have a role in uORF-mediated regulation. Given the similarities in the peptide sequence between the *Tg*ApiAT1 uORF and that of the uORF of the 'arginine attenuator peptide' (AAP) in fungi, we propose that a similar mechanism of regulation might be occurring (and we describe this further in the discussion). However, demonstrating the actual mechanism for this requires considerable further experimentation that we believe is beyond the scope of the present manuscript.

3. What wasn't clear was how the authors believe the parasite senses intracellular arginine levels? This is important and should be added to the discussion.

As noted in the previous response, our data are not conclusive on the mechanism of uORF-mediated regulation of *Tg*ApiAT1. Based on similarities between the *Tg*ApiAT1 peptide sequence at that of the fungal arginine attenuator peptide (AAP), we propose that a similar mechanism might operate (which, as we point out in the manuscript, would be a remarkable example of convergent evolution). The AAP mediates ribosome stalling on the transcript. Exactly how it does this is not known, but it has been proposed that the AAP blocks the ribosome exit tunnel in an Arg-dependent manner, possibly through Arg interacting with the peptide as it sits in the ribosome exit tunnel. We have modified the text of the discussion to state: "How the *Tg*ApiAT1 uORF mediates Arg-dependent *Tg*ApiAT1 regulation requires further experimentation. The best studied example of peptide-dependent uORF regulation is the AAP of fungi, which regulates the Arg-dependent translation of the arginine biosynthesis enzyme Arg2 [ref]. The AAP mediates ribosome stalling on the Arg2 transcript in Arg-replete conditions, possibly by blocking the ribosome exit tunnel in an Arg-dependent manner [refs]. The sequence of the *Tg*ApiAT1 uORF resembles that of the AAP (**Fig 7A**), with one of the conserved residues being critical for *Tg*ApiAT1 uORF function, and the yeast AAP being partially functional in *T. gondii* (**Fig 7B-D**). This suggests that the *Tg*ApiAT1 uORF may function in a similar manner to the AAP." (Lines 414-422)

Minor Issues:

1. Please add the ToxoDB #s for this TgApiAT1 and TgApiAT6-1 to manuscript text.

Done. These are noted the first time each gene is mentioned in the main body of the text. (Lines 75 and 200)

2. A schematic showing gene structure and position of PCR primers used in the study would be useful for readers.

We have included a schematic of the *Tg*ApiAT1 transcript as S5 Fig, indicating the positions of the 5' UTR, uORF, main ORF, start and stop codons, and other features. We refer to this figure, and describe the structure of the gene more extensively, at the beginning of Methods section where we describe the generation of genetically modified *T. gondii* strains (Lines 469-474). We subsequently refer to positions on this transcript when describing the generation of genetically modified parasites (e.g. inserting a HA upstream of the main ORF stop codon; deleting the start codon of the uORF, amplifying the 5' UTR, etc), and anticipate that this figure will aid readers in better understanding the genetic modifications.

3. The discussion regarding arginine and nitric oxide synthases was missing the fact that GRA15/ROP16, which control iNOS expression and arginine metabolism, are polymorphic but the effect of the transporter is not. Perhaps, this part of the discussion should be removed.

This is an interesting point. The reviewer is referring to studies on ROP16 (an important virulence factor in some strains of *T. gondii* which promotes expression of arginase; Saeij et al, 2006, PMID: 17170306; Butcher et al, 2011; PMID: 21931552) and GRA15 (an important virulence factor in some strains of *T. gondii* that dampens IFN-gamma-mediated upregulation of iNOS; Bando et al, 2018, PMID: 30301855). We are not suggesting that modulation of arginase and iNOS expression (and any related changes in Arg metabolism in particular cell types) are the sole "reason" for Arg-dependent *Tg*ApiAT1 regulation. It is conceivable that *Tg*ApiAT1 regulation may enable parasites to take up sufficient Arg while simultaneously modulating Arg-dependent immune responses in the host. Clearly a lot more work needs to be done in understanding the importance of host cell Arg metabolism on *T. gondii* virulence and proliferation, and we believe our study, and the luciferase-based biosensor strain we have created, provides a novel outlook and approach to these questions

We have modified the paragraph of the discussion to incorporate these points as follows: "As well as encountering different [Arg] in different organs, parasites may also encounter different [Arg] across the course of an infection. Infection by *T. gondii* parasites can lead to an upregulation of host enzymes that utilize Arg as a substrate, including arginase and nitric oxide synthases [refs]. Secreted parasite proteins such as *Tg*ROP16 and *Tg*GRA15 promote the upregulation of these enzymes in hosts [refs], and these effector proteins are important modulators of parasite virulence in some strains of *T. gondii* [refs]. Having established a means of estimating the [Arg] that parasites encounter *in vivo* (**Fig 5**), it will now be of interest to determine whether *Tg*ApiAT1 expression changes across the

course of an infection in response to the upregulation of Arg-dependent enzymes in hosts, and the role that parasite effector proteins may have in modulating host [Arg] in some strains of the parasite." (Lines 367-376)

Reviewer #3:

This study reports on a new arginine sensing system in Toxoplasma gondii that control the expression and function of the arginine transporter TgApiAT1. The authors observed that growing parasites at [arginine] ≤ 50 μM up-regulated TgApiAT1 protein abundance and arginine transport activity in a concentration-dependent manner, both in culture and in mice. Adding arginine to starved cells induced rapid degradation of TgApiAT1 to the level before starvation. The authors found that a small open reading frame within the 5'UTR of TgApiAT1 is essential for the starvation effect. Expressing TgApiAT1 ORF in a plasmid under a different 5'UTR resulted in the loss of sensitivity to low arginine content in the growth medium. The authors also reported that other essential amino acids such as lysine affect TgApiAT1 protein expression and function, but in an opposite manner then arginine. This paper describes a novel sensing system never described before in apicomplexan parasites. This sensing system resembles arginine sensing in Leishmania (reference 23).
The comparison between *T. gondii* and *Leishmania* is an interesting one, and something that we commented on briefly in our original manuscript. Both intracellular parasites regulate the abundance of their major Arg transporter, although they do so in fundamentally different ways (regulation of the Arg transporter in *Leishmania* is dependent on transcript abundance). We have added the following lines to the discussion to make these points clearer: "*L. donovani* contains a single Arg transporter (*Ld*AAP3) that is unrelated to *Tg*ApiAT1, and which is upregulated in response to a reduction of [Arg] in the growth medium [ref]. Arg-dependent changes in *Ld*AAP3 protein levels result from changes to *Ld*AAP3 transcript abundance [ref], and so is mechanistically different from the uORF-based mechanism that regulates *Tg*ApiAT1 expression in *T. gondii. L. donovani* is an intracellular parasite for part of its life cycle, inhabiting macrophages of its mammalian hosts. The presence of regulatory mechanisms in Arg transporters from two unrelated intracellular parasites suggests that tight regulation of Arg uptake may be important for the survival of intracellular pathogens in their host." (Lines 399-407)

In both, transporter protein abundance is tightly regulated by external arginine. This similarity indicates that monitoring host arginine is essential for parasite survival during infection. Moreover, nutrient sensing, especially amino acid is being recognized as key regulators of amino acid homeostasis in many biological systems. Therefore, this study is timely study that will interest many of PLoS Biology readers.
However, there are several major issues in this paper that need authors' attention.

1. Arginine concentration in mammal's serum is ~90 µM. Hence, the concentration that activates TgApiAT1 up-regulation is close to the physiological arginine concentration. This suggest that TgApiAT1 activation is not really by starvation. Typically, starvation responses initiate in at least 10-fold decrease in ligand concentration. This unusual phenomenon requires author's attention, maybe in Discussion.
We don't refer to this as being an 'arginine starvation' response. Indeed, our data from Figure 5 are consistent with the *in vivo* [Arg] that parasites encounter in different organs being within the range (~10 - 100 μM) at which we observe the greatest changes in Arg-dependent *Tg*ApiAT1 expression (nanoLUC:fLUC ratio of ~0.5 - 7). As noted in response to Comment 10 from Reviewer 1, we have expanded the discussion to include a comparison of the [Arg] in various mammalian organs to our *in vivo* data.

2. Methods lack detailed description of the starvation procedure. What is the minimal time it take parasite to respond to low arginine (below 3 hours figure 1D)? how long was a typical starvation experiment?
Regarding the minimal time it takes parasites to respond to low Arg, see our response to Comment 5 from Reviewer 1. To reiterate, we have undertaken these experiments with additional timepoints (from 30 min) and observe a rapid response to Arg depletion. Regarding the length of a typical Arg-depletion experiment, we have added the following description to the methods: "Unless otherwise indicated in the manuscript, cells were cultured in particular media conditions for two days before harvesting for subsequent assays." (Lines 433-435)
We also note the two day timeframe in our descriptions in the results (Lines 89, 99, 180)

3. Figure 1C, please calculate t50¬ value.
See our response to Comment 5 from Reviewer 1. We have not directly calculated half-life, but we do now quantify the western blotting data.

4. The effect of lysine was determined only in a medium containing 50 µM arginine. It is not clear whether lysine affects TgApiAT1 expression only in low arginine? This observation is premature and therefore this reviewer cannot understand the relevance to the overall story. Either the authors provides additional experiments to better characterize this phenomenon or, they take out this part from this paper.
The reviewer's interpretation is not quite correct. Figure 3C measures the nanoLUC:fLUC ratio in parasites grown at high (1 mM) or low (50 μM) Lys, and a range of [Arg]. We find that regulation in high vs low [Lys] is apparent at

all [Arg] tested except 10 μM (i.e. the lowest [Arg]). The relevance of this observation is that it indicates both [Arg] and [Lys] contribute to *Tg*ApiAT1 regulation (*i.e.* at a given [Lys] we observe an effect by changing [Arg], and at most arginine concentrations we observe an effect by changing [Lys]). We present evidence in Figure 4F that uptake of Arg through *Tg*ApiAT6-1 is critical for mediating Arg-dependent *Tg*ApAT1 regulation, and present a hypothesis that the Lys-dependent effect we observe (in which *Tg*ApiAT1 abundance is increased at high [Lys]) is because at high [Lys] there is decreased Arg uptake through *Tg*ApiAT6-1. This is an important conclusion of our study, and is supported strongly by the evidence we provide. We believe that the data we provide, and the conclusions we draw from these data, justifies inclusion into the manuscript

5. Results lines 95-97: why did you choose 4-fold increase as the minimum acceptable change? Why not 2-fold as do most other phosphoproteomics studies?

The 'take-home' message we are attempting to get across with these data is that *Tg*ApiAT1 is the most upregulated protein in parasites grown at low vs high [Arg]. The fold increase "cut-off" that we have applied is not important for this conclusion, and we have therefore removed the (artificial) cut-offs we included in the initial figure. In the results, we now describe these data as follows: "This revealed that although some proteins exhibited changed abundances between the two conditions, only *Tg*ApiAT1 was upregulated by >four-fold in the 50 μM compared to the 1.15 mM condition (**Fig 1E**; **S1 Table**)." (Lines 131-133)

6. Figure S1 shows time course of transport, not initial rate. Initial rate means the very beginning of transport, i.e. 1-2 minute. You may not draw a linear line from a long term transport assay. Explain how initial rate was determined.

As noted in our response to Comment 7 by Reviewer 1, we have removed the initial rate measurements and now include only the time-course uptake experiments as Figure 1F.

7. Results lines 108-110 and figure 2A: have you excluded a possible role for 3'UTR?

We cannot entirely rule out a role for the 3' UTR in *Tg*ApiAT1 regulation. However, we have demonstrated that the 5' region of *Tg*ApiAT1 is sufficient (in the absence of the *Tg*ApiAT1 3' UTR) for mediating Arg-dependent expression of nano-luciferase (e.g. Figure 2B). In the text, we state the following: "We conclude that the 5' region of the gene encoding *Tg*ApiAT1 is both *necessary* and *sufficient* to mediate Arg-dependent regulation of the *Tg*ApiAT1 protein." (Lines 169-170)

8. Figure 2B: I am puzzled by the gradual effect of external high level of arginine on arginine expression. These are higher arginine levels than normal.

Since we don't yet know how the uORF peptide functions in regulating *Tg*ApiAT1 expression, we can only speculate. As we note in the results (Fig. 7A) and discussion, the uORF-encoded peptide of *Tg*ApiAT1 resembles the so-called Arg-attenuator peptide in yeast. The model for how the yeast peptide functions is that it blocks the ribosome exit tunnel at high Arg concentrations, thereby causing ribosome stalling (Bushan et al, 2010, PMID: 20932481). Increasing [Arg] beyond 'physiological' levels in the yeast system (e.g. to 5 mM) leads to a greater increase in ribosome stalling (Fang et al, 2004, PMID: 15020769). So it is conceivable that, like in the yeast system, increasing [Arg] beyond physiological levels further increases ribosome stalling and associated repression of the downstream main ORF. Given that this is speculation, we have not discussed this extensively in the manuscript. We state the following:

"We demonstrated that Arg-dependent regulation of *Tg*ApiAT1 is mediated by a uORF in the *Tg*ApiAT transcript (**Figs 6-7**). uORFs appear to be abundant in *T. gondii* transcripts [ref], and the uORF of *Tg*ApiAT1 represents the first characterised example of a functional uORF in these parasites [ref]. Our data indicate that the peptide encoded by the uORF plays a role in the Arg-dependent regulation of *Tg*ApiAT1 expression (**Fig 7**). This is one of only a few known cases in which the peptide of a uORF appears to be critical for regulating translation of the downstream main ORF [ref]. How the *Tg*ApiAT1 uORF mediates Arg-dependent *Tg*ApiAT1 regulation requires further experimentation. The best studied example of peptide-dependent uORF regulation is the AAP of fungi, which regulates the Arg-dependent translation of the arginine biosynthesis enzyme Arg2 [ref]. The AAP mediates ribosome stalling on the Arg2 transcript in Arg-replete conditions, possibly by blocking the ribosome exit tunnel in an Arg-dependent manner [refs]. The sequence of the *Tg*ApiAT1 uORF resembles that of the AAP (**Fig 7A**), with one of the conserved residues being critical for *Tg*ApiAT1 uORF function, and the yeast AAP being partially functional in *T. gondii* (**Fig 7B-D**). This suggests that the *Tg*ApiAT1 uORF may function in a similar manner to the AAP. Given that *T. gondii* and fungi are separated by ~1.5 billion years of evolution, and that the *Tg*ApiAT1 uORF peptide appears restricted to *T. gondii* and its closest relatives, a conserved function between these uORFs would represent a remarkable example of convergent evolution." (Lines 408-425)

9. Results line 114: "we grew…" please explain how the experiment was carried out.

We have modified the text in the results section to clarify this further: "We cultured this 'dual reporter' strain at [Arg] ranging from 10 μM to 5 mM for 40-42 hr and measured nanoLUC- and fLUC-dependent luminescence." (Lines 159-160). We have also added the following text to the Figure 2B legend to specify this (change underlined): "nanoLUC:fLUC ratio in a parasite strain expressing nanoLUC from the *Tg*ApiAT1 5' region (*apiAT1*

5'-nanoLUC) and fLUC from the α-tubulin 5' region (*tub* 5'-fLUC), and grown at a range of [Arg] for 40-42 hr." (Lines 963-965)

10. Lines 137-139: sentence not clear

We have changed the word "higher" to "greater". This now reads: "At all but the lowest Arg concentration tested (*i.e.* 10 μM), the nanoLUC:fLUC luminescence ratio measured in parasites grown at high [Lys] was greater than that measured in parasites grown at low [Lys] (**Fig 3C**)" (Lines 183-185)

11. The cells in figure 3 are in 50 µM arginine. Why we do not see the arginine transporter in this concentration? Explain in text.

We are unsure what the reviewer means here. We show in this figure that *Tg*ApiAT1 abundance is modulated by changes in the concentration of a range of nutrients. We chose 50 μM Arg for these studies because this represents an 'intermediate' [Arg], where the (major) contribution of [Arg] to the regulation will not 'overwhelm' the effects of these other nutrients. For example, we shown in Figure 3C that [Lys] has no effect on *apiAT1 5'*-nanoLUC expression when parasites are grown at 10 μM Arg, whereas there is ~5-fold less expression of nanoLUC in 50 μM Lys than in 1 mM Lys when parasites are grown in 50 μM Arg.

12. Line 142 and figure 3: in these experiments no indication for how long cells were exposed to each of these nutrients.

See our response to Comment 2 (above). We now specify in the methods section that parasites were cultured in each media condition for two days before harvesting, and we also mention this in relevant parts of the results section.

13. Line 156: this is an illegal statement. You may not include unpublished data in results

We have recently posted our manuscript on *Tg*ApiAT6-1 onto a pre-print server (Fairweather *et al.*, 2021, see link in our response to Comment 4 by Reviewer 1), and have cited this at relevant points in the manuscript. Since this posted manuscript includes a description of the ATc-regulatable strain of *Tg*ApiAT6-1, we have removed the description in the present manuscript (previously Figure S4).

14. Figure 7C: This reviewer is not convinced by data as changes occurred only at very high arginine concentrations.

The differences are small but reproducible (26-34% decrease in the nanoLUC:fLUC ratio in parasites cultured in medium containing 50-1000 μM Arg, and a ~10-fold decrease in the 5000 μM Arg condition, compared to parasites cultured in 10 μM Arg). As we note in describing these experiments, one possibility for the small differences is the length of the yeast uORF. When we extend the yeast uORF to be the same length as the *Tg*ApiAT1 uORF, the extent of regulation is increased (Figure 7D). To be consistent with the rest of the manuscript, we have presented the y axis on a log10 scale, which may make the differences 'appear' smaller to the reviewer than they are. We believe that we are appropriately cautious in interpreting these data, stating that "We observed a small but significant decrease in the nanoLUC:fLUC ratio with increasing [Arg], most noticeably at the highest [Arg] tested." (Lines 316-317)

15. Discussion line 290: see comment 12

We are unsure what the reviewer means here. Perhaps they mean comment 13? As noted earlier, we have posted our manuscript on *Tg*ApiAT6-1 onto a pre-print server. If they do mean comment 12, see our response to Comment 2 (above). We now specify in the methods section and relevant sections of the results that parasites were cultured in each media condition for two days before harvesting.

16. Discussion is too long. It mostly repeats results with minimal debates on the outcome and global meaning of the observations made in Results.

As noted in our responses to other comments, we have modified the discussion to consider various points raised by the reviewers. Specifically we:

- Discuss the roles of TgApiAT1 and TgApiAT6-1 in modulating Arg uptake by the parasite *in vivo* (Lines 338-350)
- Expand the discussion to mention the [Arg] that parasites likely encounter in different organs, and compare this to our *in vivo* data (noting the concurrence between our findings and previous studies in the [Arg] that parasites encounter in liver and kidneys). We also mention the role that other nutrients may have on *Tg*ApiAT1 expression *in vivo* (Lines 351-366)
- Mention the role of parasite proteins in modulating the expression of host enzymes involved in arginine metabolism, and how this may related to Arg-dependent TgApiAT1 regulation (Lines 367-376)
- We expand our comparison between *Tg*ApiAT1 regulation and regulation of the Arg transporter of *Leishmania donovani*, noting that these unrelated intracellular parasites both exhibit tight control over Arg uptake, albeit by fundamentally different mechanisms (Lines 399-407)
- We expand on the possible mechanism by which parasites sense Arg (Lines 408-425)

17. Figure 5B: Details are missing from legend; what day were they harvest? How did the authors change and then monitored arginine level in mice.
The reviewer mis-understands this graph, possibly because our initial descriptions of these data may not have been clear. The purpose of this figure was to provide a comparison between the *in vivo* data and the *in vitro* data. The graph indicates that the concentrations of [Arg] that parasites are predicted to encounter *in vivo* are within the range at which we observe Arg-dependent regulation of *Tg*ApiAT1 *in vitro*.

As we state in the figure legend, the x axis represents the *in vitro* data shown in Figure 2B. We have attempted to be clearer about this in the figure legend, which now reads (key changes underlined): "The mean nanoLUC:fLUC luminescence ratios of *apiAT1* 5'-nanoLUC:*tub* 5'-fLUC parasites harvested from various mouse organs and peritoneal cavity in (**A**) shown as dashed lines from the y axis, mapped onto the nanoLUC:fLUC luminescence ratios of *apiAT1* 5'-nanoLUC:*tub* 5'-fLUC parasites grown *in vitro* at a range of [Arg] (solid black line; these data are redrawn from the data depicted in Fig 2B)." (Lines 1011-1017)

18. Figure 5B: How can you control mice arginine in the blood during infection?
See our response to the previous comment.

In addition to comments from the reviewers at PLOS Biology, we also received feedback from Chhaminder Kaur on a version of the manuscript that we posted onto BioRxiv. We would like to take this opportunity to respond to those comments as well (and note that a modified version of these responses were posted onto BioRxiv).

1.Figure 1B: For bradyzoite induction at high pH, the methods section clearly states that the parasites were maintained in a media with high pH for 6 days. However, it is not clear for how many days the parasites have been starved of argininebefore the experiment is performed. This data point will be crucial in eliminating the hypothesis that the bradyzoite induction did not happen due to less time given for them to differentiate.

We cultured Type II parasites in medium containing a range of [Arg] for two days and we now specify this in the text. The commenter is correct that this may not be long enough to observe Arg-induced bradyzoite induction (although it is curious that we don't see upregulation of BAG1, an earlier marker for bradyzoite differentiation, in the two days at lower [Arg]). Testing whether low [Arg] induced bradyzoite differentiation was, however, not the intention of the experiment. Rather, we were interested in whether inducing bradyzoite differentiation, which we do by treating parasites at high pH for six days, causes an increase in ApiAT1 abundance. We don't see this, from which we conclude that the upregulation of ApiAT1 is not part of any general bradyzoite-specific response, but rather a specific effect of Arg depletion. We have modified this paragraph to make clearer how we performed the experiment (key changes underlined below), and to clarify that our data do not test whether TgApiAT1 is involved in bradyzoite differentiation (also underlined):

"Low [Arg] conditions have been linked to formation of the latent bradyzoite stage of *T. gondii* [ref]. We HA-tagged *Tg*ApiAT1 in Type II Prugniaud strain *T. gondii* parasites, which readily form bradyzoites, and cultured parasites for two days at a range of initial [Arg] in the culture medium. As for Type I parasites, expression of *Tg*ApiAT1-HA_3 increased with decreasing [Arg] (**Fig 1B**). To test whether *Tg*ApiAT1-HA_3 abundance changed upon bradyzoite differentiation, we induced bradyzoite differentiation by incubating parasites for six days in high pH conditions in a medium containing 1.15 mM Arg. We observed increased expression of the bradyzoite-specific antigen *Tg*BAG1 and a bradyzoite-specific green fluorescence protein (GFP), and decreased expression of the tachyzoite specific antigen *Tg*SAG1, verifying that bradyzoite differentiation had occurred (**Fig 1B**). Notably, we observed no induction of *Tg*ApiAT1-HA_3 expression in these conditions (**Fig 1B**). These data indicate that *Tg*ApiAT1 regulation is not related to the general bradyzoite differentiation response of the parasite,_and that Arg-dependent *Tg*ApiAT1 regulation occurs in both Type I and Type II strains of *T. gondii*. The short timeframe over which we cultured parasites in depleted [Arg] means that our data do not rule out the possibility that Arg depletion causes bradyzoite differentiation, or rule out a role for *Tg*ApiAT1 in mediating bradyzoite differentiation." (Lines 97-112)

2.Previous studies show that arginine starvation leads to the conversion of the fast-replicating tachyzoites to the slow-growing bradyzoites (Fox et al., 2004). This would mean that the absence of the arginine transporter, TgApiAT1 would lead to the formation of bradyzoites. However, the authors show that the presence of another cationic amino acid transporter, TgApiAT6-1 can also transport arginine with low affinity. This shows that the parasite has a fail-safe mechanism in case TgApiAT1 is not expressed due to other unknown reasons. The presence of a fool-proof mechanism suggests that arginine concentration plays a vital role in deciding the stage of the parasite.
The interplay between *Tg*ApiAT1 and *Tg*ApiAT6-1 is fascinating. The commenter is correct that evolving two transporters with different affinities for Arg - a highly selective, regulatable Arg transporter (TgApiAT1) and a

general cationic transporter (TgApiAT6-1) with lower affinity for Arg - enables these parasites to modulate Arg uptake in intracellular environments where they might encounter differing concentrations of Arg (and other cationic amino acids). We draw these points together in a model that we present in the Fairweather et al manuscript posted on BioRxiv (see Fig 7 of that manuscript).

3.Gene IDs of TgApiAT1 and TgApiAT6 are not mentioned.

The ToxoDB gene IDs for *Tg*ApiAT1 is TGGT1_215490 and for *Tg*ApiAT6-1 is TGGT1_240810. As noted in response to minor comment 1 from Reviewer 2, the text has been modified to now include these. The nomenclature and evolutionary relationships between the various ApiAT family proteins in apicomplexans is described in an earlier study from our group (PMID 30742695).

4.In Plasmodium falciparum, PfAAT1 (PF3D7_0629500) is the homologous arginine transporter. The transcript of this amino acid transporter harbours 11 uORFs in its leader sequence. Our analysis shows that these uORFs encode for peptides that do not share any similarity with the conserved yeast arginine attenuator peptide (AAP). It is possible that P. falciparum might be employing another mechanism to regulate the expression of this gene.

We previously characterised a plasma membrane-localised general cationic amino acid transporter in *Plasmodium berghei* parasites (PBANKA_020830 - initially termed PbNPT1, although we now call this PbApiAT8) in an earlier study from 2017 (PMID 28205520). *Pb*ApiAT8 is orthologous to *Pf*ApiAT8 (PlasmoDB gene ID PF3D7_0104800) but is probably not a direct homologue of TgApiAT1 (functionally, it is more similar to TgApiAT6-1). PbApiAT8 is critical for Arg uptake into asexual stage parasites and for the progression of parasites into the insect stages of the parasite life cycle — see our 2017 study and also the following studies from other groups for more details: Boisson et al (PMID 21752119) and Kenthirapalan et al (26796412). The PfAAT1 protein mentioned by the commenter is not an ApiAT family protein, and we've not investigated it in any detail. But it will be of interest to determine how widespread nutrient-dependent uORF-mediated regulation of solute uptake and biochemical pathways is in these parasites. Such post-transcriptional modes of regulation enable parasites to respond rapidly to changes in the nutrient status of their environments as they enter different tissues/organs/hosts.
